# Pressure driven rotational isomerism in 2D hybrid perovskites

**DOI:** 10.1038/s41467-023-36032-y

**Published:** 2023-01-25

**Authors:** Tingting Yin, Hejin Yan, Ibrahim Abdelwahab, Yulia Lekina, Xujie Lü, Wenge Yang, Handong Sun, Kai Leng, Yongqing Cai, Ze Xiang Shen, Kian Ping Loh

**Affiliations:** 1 Division of Physics and Applied Physics, School of Physical and Mathematical Sciences, Nanyang Technological University, Singapore, 637371 Singapore; 2 Joint Key Laboratory of Ministry of Education Institute of Applied Physics and Materials Engineering, University of Macau, Macau, China; 3 Department of Chemistry, National University of Singapore, Singapore, 117543 Singapore; 4 Centre for Disruptive Photonic Technologies, the Photonics Institute, Nanyang Technological University, Singapore, 637371 Singapore; 5 Center for High Pressure Science and Technology Advanced Research, (HPSTAR), Shanghai, 201203 P.R. China; 6 Department of Applied Physics, The Hong Kong Polytechnic University, Hung Hom, Kowloon, Hong Kong China

**Keywords:** Materials science, Nanoscale materials

## Abstract

Multilayers consisting of alternating soft and hard layers offer enhanced toughness compared to all-hard structures. However, shear instability usually exists in physically sputtered multilayers because of deformation incompatibility among hard and soft layers. Here, we demonstrate that 2D hybrid organic-inorganic perovskites (HOIP) provide an interesting platform to study the stress–strain behavior of hard and soft layers undulating with molecular scale periodicity. We investigate the phonon vibrations and photoluminescence properties of Ruddlesden–Popper perovskites (RPPs) under compression using a diamond anvil cell. The organic spacer due to C4 alkyl chain in RPP buffers compressive stress by tilting (*n* = 1 RPP) or step-wise rotational isomerism (*n* = 2 RPP) during compression, where *n* is the number of inorganic layers. By examining the pressure threshold of the elastic recovery regime across *n* = 1–4 RPPs, we obtained molecular insights into the relationship between structure and deformation resistance in hybrid organic-inorganic perovskites.

## Abstract (web summary)

Multilayers comprising alternating soft and hard layers offer enhanced toughness compared to all-hard structures. Here authors reveal how the hard and soft components in Ruddlesden–Popper perovskites work cooperatively to resist deformation under pressure, informing the design of alternating superlattices for engineering applications.

## Introduction

Two-dimensional (2D) Ruddlesden-Popper perovskites (RPPs) consist of alternatively stacked layers of soft organic layers and rigid inorganic layers with highly tunable optoelectronic properties^1,2^. The organic layers provide the dielectric and quantum confinement of the inorganic layers, giving rise to large exciton binding energies and high oscillator strength^3–5^. From a structural mechanics point of view, the inorganic layers serve as mechanical brace to support spring-like molecular layers, forming a “natural” undulating hard-soft system. In the realm of mechanical engineering, such a hard-soft multilayer system is highly sought after for its high plastic deformation resistance and increased fracture hardness, but requires a complex fabrication process. The undulating layers create bands of tensile and compressive stress that is different from that of single-component layers, thus research efforts on inorganic hard-soft multilayers are centered on engineering structure and compositional gradient to enhance the mechanical properties of materials. The general chemical formula for lead halide 2D RPPs is (RNH_3_)_2_M_n−1_Pb_n_X_3n+1_ (*n* represents the number of inorganic layers), where PbX_4_^2-^ is an inorganic perovskite layer of corner-sharing metal halide octahedra, RNH_3_^+^ is a long-chain alkylammonium organic molecular layer, such as butyl ammonium (BA), and M is a smaller cation filled into the 12-fold coordinated holes wrapped by the PbX_6_ octahedra, such as CH_3_NH_3_^+^ (MA^+^)^6–8^. The long-range structure of the organic array is a result of balancing local strain induced by methyl ammonium ions on the inorganic cage, hydrogen-bonding and electrostatic forces between the cationic head group of the organic molecule and the metal halide framework^9,10^. Thanks to the rigid-/soft-layer alternated stacking structure of 2D layered perovskites, the rigid inorganic framework can template the conformation of the soft organic molecule confined in the organic-inorganic atomic interface^11^. In the ground state of C4 alkyl chain, each CH_2_ moiety adopts the trans-trans (tt) conformation to avoid steric repulsion. Under compression, it can be expected that the lamellar layers will contract and the strain in the metal halide bonds will affect optical and electronic properties^12–21^. However, little is known about the conformational change of the organic cations and the plastic deformation or strain hardening process when the organic molecules re-orientate under pressure, this is because X-ray diffraction is mostly sensitive to the heavier inorganic components.

Here, using a combination of in situ Raman and Photoluminescence (PL) spectroscopy, we investigate how compressive and tensile strain develops in the organic and inorganic components in (BA)_2_MA_n−1_Pb_n_I_3n+1_ (*n* = 1-4) during compression. Combining experiments and density functional theory (DFT), we observed that *n* = 1 and *n* > 1 exhibit markedly different mechanisms of compressive strain under pressure. In *n* = 2 RPP, under compression, the trans-trans (tt) structure of the butyl ammonium cation gradually transforms into the trans-gauche (tg) and gauche-gauche (g^+^g^-^ and g^+^g^+^) conformations via C-C and C-N bond rotations, along with tensile strain of the inorganic slabs in both *a* and *b* axial directions. In contrast, for *n* = 1 RPP, tilting of the linear organic cation is along with in-plane shift in one direction (*b* axis), resulting in orthorhombic-to-monoclinic phase change under compression. *n* = 1 RPP shows remarkable elastic recovery ability due to the flexibility of organic cation that acts synergistically with the octahedral tilt of the inorganic cages. Our study shows how differing alternation of soft and hard layers allows compressive and tensile stress to evolve differently across *n* = 1 to *n* = 4 RPPs, which provides the essential experimental basis for understanding stress-strain behavior in nanometer scale multilayer hard-soft superlattices.

## Results

To lay the groundwork for understanding the complex structural changes in the organic and inorganic parts of the hybrid perovskites upon hydrostatic compression, it is beneficial to organize the paper by discussing the changes revealed by first-principles calculations first, followed by comparison with what was observed in the experiments.

### Theoretical calculations of lamellar contraction

**Fig. 1:**
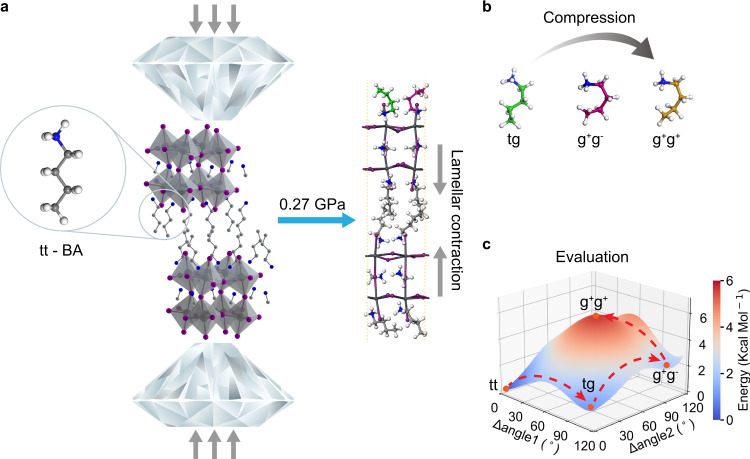
Rotational isomerism of BA molecules during compressive stress of *n* = 2 RPP. **a** Schematic showing diamond anvil cell and the *n* = 2 RPP crystals with linear tt-BA organic chains. **b** DFT simulation of lamellar contraction and the step-wise rotational isomerism. **c** DFT simulated energy landscape of the phase space of BA isomers (tt, tg, g^+^g^-^ and g^+^g^+^), and their transformation process is marked by a dashed red line.

Figure 1a depicts a unit cell of *n* = 2 RPP. Two layers of interdigitated linear organic BA molecules alternately stack with two layers of Pb_2_I_7_^3-^ inorganic slabs. The unit cell height of ~ 2.1 nm is determined by atomic force microscopy (AFM)^22^. BA organic molecules in tt conformation dominate the large interlayer spaces, while MA ions occupy the inter-octahedral voids (Supplementary Fig. 1a). In the case of *n* = 1 RPP, there are no MA ions and one layer of PbI_4_^2-^ inorganic slab is alternatively stacked with two layers of BA molecules (Supplementary Fig. 2).

To simulate hydrostatic compression, a two-step strategy (see Supplementary Discussion for details) was adopted with the bi-layer RPP sample being sandwiched between two diamond slabs (Fig. 1a and Supplementary Fig. 1a). Under compression, the lamellar distance contracts. In the low pressure range (<~ 3 GPa), compressive stress is accommodated mainly by the deformation of the organic layers. At 0.27 GPa, the lamellar contraction is achieved by rotation of the tt isomer of BA to tg (green color) and g^+^g^-^ (purple color) conformers on the surface of RPP, which can be accomplished by rotating along the C-C/C-N chain (Fig. 1b). The rotational energy landscape of BA (Fig. 1c) shows that the linear tt isomer is the global minimum state of the BA molecule, which can transform into the tg and g^+^g^-^ conformers across a barrier of ~ 1.1-2.8 and ~ 3.2 Kcal/Mol, respectively, as listed in Table S1. Relative to the initial tt state with its energy set to zero as a reference point, the g^+^g^+^ conformer has a higher energy of 4.5 Kcal/Mol (Table S1), therefore it is unstable and  transforms into other conforms readily. The tg isomers are classified into the tg-kind1 and tg-kind2 according to the asymmetry of tail and head, as shown in the Supplementary Fig. 6, they share similar energy but opposite dihedral angles. The rotation of the organic cations allows the Pb_2_I_7_^3-^ inorganic cage to resist deformation at compressive pressure below ~ 3 GPa (Supplementary Fig. 1a). In contrast, the BA molecules are transformed into the BA conformers with the comparable energies (Table S1), i.e., tg and g^+^g^-^ isomers are energetic local minimum states, leading to their ambient stability. The ability to generate new rotational conformers in RPP by compression is subsequently verified in our experiments shown later.

**Fig. 2:**
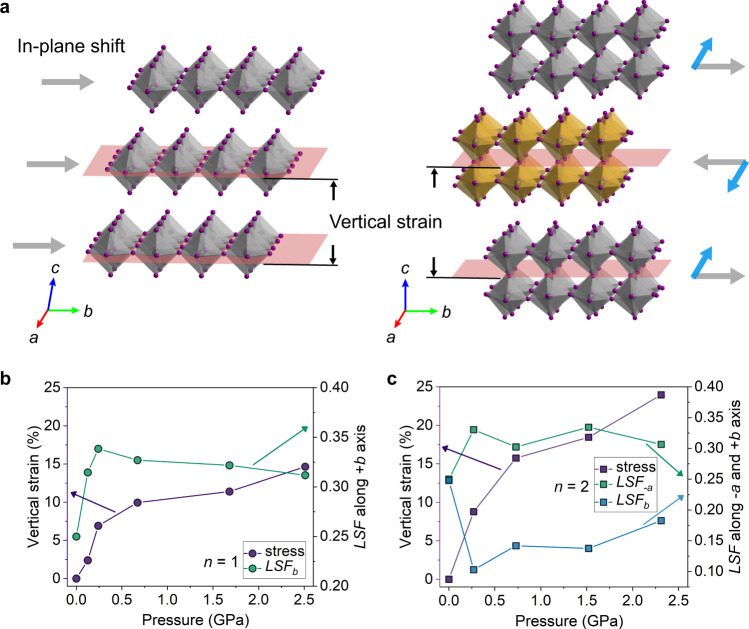
Distinctive pressure responses of *n* = 1 and *n* = 2 RPP. **a** Two generic types of layer-shift mode: Γ_5_^+^ for *n* = 1 and M_5_^-^ (*a*, *b*) for *n* = 2 RPP. **b**, **c** Pressure-dependent in-plane layer shift factor (*LSF*) and vertical strain for *n* = 1 and 2 RPPs. Source data are provided as a Source Data file.

Compression results in the layer-to-layer shift of the bi-layer inorganic slabs in *n* = 2 RPP along *- a* and *+ b* axes, as represented by the blue and grey arrows in Fig. 2a (right side), which is related to a ${M}_{5}^{-}(a,\, b)$ mode^23^. Such an interlayer shift maintains *n* = 2 RPP in the orthorhombic symmetry, accompanied by the conformational change of BA molecules without tilting under pressure.

Interestingly, we observed pronounced differences in the pressure response of *n* = 1 RPP compared to its higher homologues, as illustrated in Fig. 2. For *n* = 1 RPP, the linear BA molecules tilt without undergoing conformational change till 7.36 GPa (Supplementary Fig. 2a). The molecular tilting drives the adjacent inorganic layer to shift in the same direction along *+b* axis, as represented by the grey arrows in Fig. 2a (left side), which is related to a ${\Gamma }_{5}^{+}$ mode^23^. The layer shift decreases the initial stacking offset and therefore the crystal undergoes a phase transition to the monoclinic class^24^. Such pressure-induced layer-to-layer shift is however absent in *n* = 2 RPP ( < ~3 GPa). The detailed structural evolution of *n* = 1 and *n* = 2 RPP with pressure is presented in the Supplementary Fig. 4.

### In-plane shift

The discontinuity in the *c* direction of 2D RPPs allows an in-plane layer shift between the adjacent inorganic layer, which can be evaluated by a layer shift factor (*LSF*)^25^. Here, using the lower inorganic layer as the reference, the relative coordinate deviation of the upper layer along the *a* axis is calculated as:

1 $${{LSF}}_{{{{{{\rm{a}}}}}}}={\sum} _{{n}}(\bar{{l}_{1}}-\bar{{l}_{2}})/n{L}_{{{{{{\rm{a}}}}}}}$$

Where $\bar{{l}_{1}}-\bar{{l}_{2}}$ is one of the in-plane projected vectors of Pb atoms pair (${l}_{1}$, ${l}_{2}$) from the first and second layers in the $\sqrt{2}\,\times \sqrt{2}$ supercell metric, ${L}_{{{{{{\rm{a}}}}}}}$ is the corresponding lattice parameter. The standard RPPs have an initial *LSF* value of 0.25 on both *a* and *b* directions^25^, referring to the Supplementary Fig. 4 for the top views of layer shift between *n* = 1 and 2 RPPs in the low-pressure region (<~ 3 GPa, where the lamellar contraction is dominated by BA organic layers). The calculated *LSF* are represented by the green filled circles for *n* = 1 in Fig. 2b and green /blue filled cubes for *n* = 2 in Fig. 2c. The *n* = 1 RPP has a maximum *LSF*_*b*_ value of ~ 0.34 at 0.24 GPa, while *n* = 2 RPP accommodates *LSF*_*a*_ of ~ 0.33 and *LSF*_*b*_ of ~ 0.10 along *- a* and *b* directions, respectively. The *n* = 1 RPP has an average *LSF*_*b*_ value of ~ 0.32 ± 0.01 within pressure of 2.51 GPa, while *n* = 2 RPP accommodates an average *LSF*_*-a*_ value of ~ 0.32 ± 0.01 and *LSF*_*b*_ value of ~ 0.14 ± 0.03 along *- a* and *b* directions within pressure of 2.31 GPa.

Here, the vertical strain is defined as the relative interlayer distance change comparing that of the initial layer distance, following with this formula:

2 $${\triangle }_{d}=({d}_{{{{{{\rm{p}}}}}}}-{d}_{0})/{d}_{0}$$

where the ${d}_{{{{{{\rm{p}}}}}}}$ and ${d}_{0}$ are the inorganic interlayer distance at ${{{{{\rm{p}}}}}}$ GPa and $0$ GPa, respectively. According to our calculations, the tilting of BA cations in *n* = 1 RPP results in a vertical strain of ~ 14.6 % within pressure of 2.51 GPa (violet filled circles in Fig. 2b), while the rotation isomerism of BA cations in *n* = 2 RPP results in ~ 23.9% vertical strain within pressure of 2.31 GPa (violet filled cubes in Fig. 2c).

### Compressibility and octahedral tilt

**Fig. 3:**
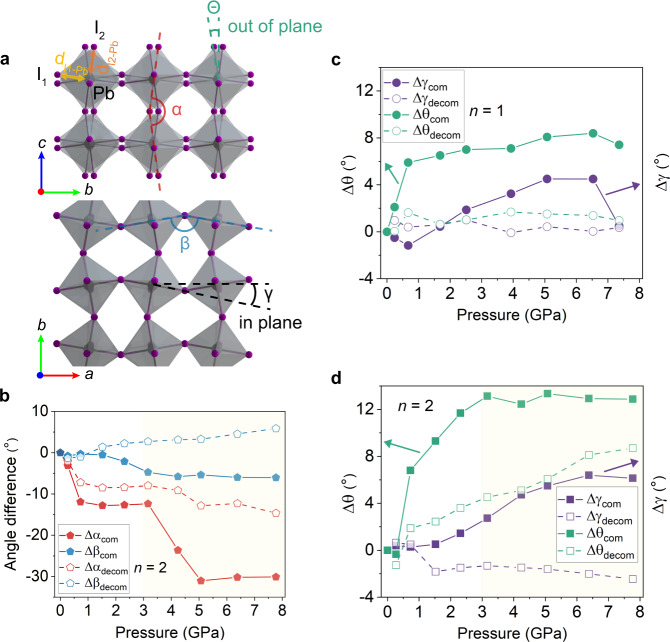
Evolution of the inorganic lattice parameters of *n* = 1 and *n* = 2 RPP under compression and decompression. **a** Schematic of the *n* = 2 RPP 2D Pb-I octahedral cages, highlighting the apical and equatorial angle α and β, and the out-of-plane and in-plane octahedral tilt angle ɵ and γ, and bond length d_I1-Pb_ and d_I2-Pb_, respectively. **b** Evolution of Δα and Δβ in *n* = 2 under compression and decompression. Evolution of the Δɵ and Δγ in *n* = 1 RPP (**c**) and *n* = 2 RPP **d** under compression and decompression. The yellow-shaded area in **b**, **d** represents the irreversible deformation region. Source data are provided as a Source Data file.

To track the structural deformation and recoverability of the inorganic sublattice during compression-decompression cycles, we analyze several key structural parameters that represent the in-plane and out-of-plane components of the *n* = 2 RPP Pb-I octahedral cage (Fig. 3a), namely, anionic cage angles (β and α), octahedral tilting angles (γ and ɵ) and Pb-I bond lengths (d_I1-Pb_ and d_I2-Pb_). To track the deviation from the initial angles at 0 GPa after compression, we monitor the difference in angles ∆θ between the final and initial state.

Under compression, *n* = 2 RPP undergoes lamellar contraction by the rotational isomerism of the softer BA cations, this allows the harder Pb-I lattice to remain in the elastic regime when the pressure is less than ~ 3 GPa. The apical angle α linking two octahedral cages serves as a pivot and bends by up to ~ 12.4° during vertical compression, and unbends a little bit more after decompression (red symbols in Fig. 3b). The sudden drop in α beyond ~ 3 GPa reflects the pressure threshold in the *n* = 2 system, beyond which irreversible deformation occurs. On the other hand, the change in the equatorial angle β subtends by Pb-I-Pb in the *ab* plane (blue symbols in Fig. 3b) reflects the intra-layer compressive strain, ∆β gives a negative difference because the compressed angle is smaller than the angle before compression, and it shows a distinct drop beyond the same pressure threshold as ∆α. Besides, the structural distortion of the inorganic sublattice can also be revealed by the octahedral tilt, where the out-of-plane tilt is much more pronounced than the in-plane tilt (solid symbols in Fig. 3d).

In the case of *n* = 1 RPP, lamellar contraction occurs by the tilting of the organic cation (Supplementary Fig. 2a). The maximum value of the in-plane octahedral tilt (Δγ = 4.5°) and the out-of-plane tilt (Δɵ = 8.4°) (solid symbols in Fig. 3c). These two tilt angles seem to display good recoverability after decompression as compared to those of *n* = 2 RPP, as represented by the hollow symbols in Fig. 3c. It suggests that *n* = 1 RPP resists vertical compression by the combination of the tilting of organic cation as well as octahedral tilts of the inorganic sublattice. An important difference is that the tilted organic cations in *n* = 1 RPP can be untilted upon decompression (Supplementary Fig. 2b), whereas the rotation isomers in *n* = 2 RPP are locked and cannot be reverted to the original isomers upon decompression (Supplementary Fig. 1b). The better recovery of bond lengths in *n* = 1 after decompression can further prove the distinct pressure response of BA molecule between *n* = 1 and *n* = 2 RPP, as shown in the Supplementary Fig. 5.

To better understand the organic BA isomerism and inorganic octahedral tilting under finite temperature and pressures, ab initio molecular dynamics simulations (AIMD) were performed for both *n* = 1 and 2 RPPs. The free energies for isomerism and lattice tilting were deduced from the probability distribution of inner molecular angles of the BA molecule and azimuthal angles of the sublattice (Supplementary Figs. 7–9). For the organic part, as shown in the Supplementary Fig. 7c-g, the barrier of the inner molecular rotation of BA within RPP layers from tt to tg is found to be ~ 0.6-0.9 Kcal/Mol, which is slightly lower than the barrier of ~ 1.1-2.8 Kcal/Mol obtained from DFT calculations (Fig. 1c). More importantly, the stable tg BA isomer is formed at 0.5 GPa in *n* = 2 RPP, and a lower energy barrier is obtained upon further compression, e.g., ~ 12.5-25% in decrease at 3.0 GPa (Supplementary Fig. 7e-g). As a comparison, there is no new stable isomers formed in *n* = 1 RPP within 10 GPa, and compression will increase the rotation barrier (Supplementary Fig. 7c, d). For the inorganic part, the Pb-I skeleton shows strong anharmonicity within the dynamic tilting process, as shown in the Supplementary Figs. 8 and 9, and the local skeleton distortion induces a remarkable energy double-wall potential along the tilting direction. At 5.0 GPa, the more asymmetric potential in *n* = 2 RPP indicates its stronger octahedral distortion induced by the strong hydrogen bond interaction between the Pb-I skeleton and BA molecules. The AIMD calculations further verify the distinct pressure response for *n* = 1 and *n* = 2 RPP.

### Raman spectroscopic evidence of rotated BA conformers

Raman spectroscopy is a powerful tool used to characterize the vibrations and rotations of organic molecules in organic-inorganic hybrid perovskites during the pressurization process^26,27^. The single crystal of *n* = 2 RPP was synthesized through a temperature-programmed crystallization method^28,29^, and the exfoliated flakes were loaded into the diamond anvil cell (DAC) for in situ high pressure Raman measurement. NaCl was used as the pressure transmitting medium (PTM) to obtain a clean Raman signal of the organic molecular vibrations.

**Fig. 4:**
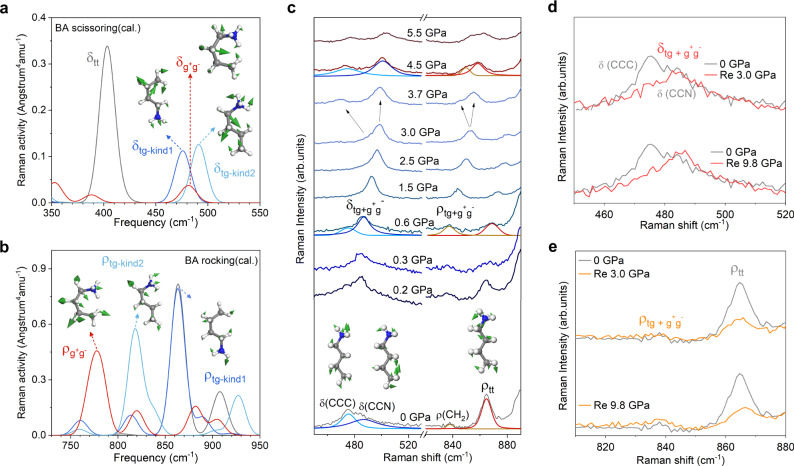
Raman characterization of the BA tg- and g^+^g^-^- conformers. **a**, **b** Calculated Raman spectra for the BA scissoring and rocking modes in the tt, tg and g^+^g^-^ structures. **c** Typical Raman spectral evolution of *n* = 2 RPP with increasing pressure. **d**, **e** Raman spectral comparison of RPP sample before and after compression. δ and ρ refers to scissoring and rocking vibrations. The grey, dark (light) blue and red lines in (**a**, **b**) represent BA vibrations in the tt, tg and g^+^g^-^ conformations, respectively. Insets represent the corresponding vibration patterns of BA isomers, green arrows on the atoms indicate the vibrational directions and relative amplitude of displacements in the given Raman modes. Source data are provided as a Source Data file.

As a reference, we have calculated the Raman spectra of BA^+^ gas molecule in the tt, tg-kind1 (tg-kind2) and g^+^g^-^ conformations, as represented by the grey, dark (light) blue and red solid lines in Fig. 4a, b (See the detailed peak assignment in the Supplementary Discussion). The corresponding calculated vibrational modes are listed in Table S2 and S3. Besides, to test the effect of Pb-I inorganic lattice on the BA vibrational frequencies, a phononic calculation was also performed for BA isomers in *n* = 2 RPP structure, as shown in Table S4. The Raman results show a similar shift behavior during the conformational change similar to that of BA in gas form, thus, the following discussion of Raman results is based on BA^+^ gas molecules.

Our measured in situ high-pressure Raman spectra of *n* = 2 RPP under compression, as shown in Fig. 4c, reproduces largely the Raman peak evolutions during the trans to gauge conformational change of the BA^+^ gas molecules. The measured Raman bands at 0 GPa in the frequency region of 450 − 890 cm^−1^ are the skeletal vibrational modes of BA molecules in the tt conformation, as represented by grey solid line in Fig. 4a–c. The Raman bands appearing at ~ 475.3 and 484.7 cm^−1^ belong to the scissoring modes of tt-BA, i.e., δ(CH_2_-CH_2_-CH_3_) (δ(CCC)) and δ(CH_2_-CH_2_-NH_3_^+^) (δ(CCN)), which are associated CH^3^ and HN^3+^ stretch in opposite directions (Fig. 4a, Table S2, and the vibration modes shown in the inset of Fig. 4c). The observed characteristic band at ~ 864.7 cm^−1^ belongs to the rocking mode of tt-BA, i.e., ρ_tt_. Besides, there is a very weak band at ~ 837.2 cm^−1^ originating from the rocking of CH_2_ (Fig. 4b, Table S3, and the vibrational mode shown in the inset of Fig. 4c). Upon compression at 0.2 GPa, the Raman spectrum shows distinct changes. First, the δ(CCC) and δ(CCN) bands almost merge into one band at ~ 487.2 cm^−1^, which is the spectrally overlapped scissoring modes of BA in the trans-gauche and gauche-gauche form, i.e., δ_tg_ and δ_g+g-_, respectively (Fig. 4a). Besides, the intensity of ρ_tt_ weakens (Raman inactive-like) under compression, while the intensity of the Raman band at ~840 cm^−1^ is enhanced due to the formation of Raman-active tg and g^+^g^-^ BA conformers (Fig. 4b). From 0.6 to 3.0 GPa, the characteristic scissoring and rocking modes of tg and g^+^g^-^ BA conformers show a continuous blue shift (Supplementary Fig. 10b, c), which reflects lamellar contraction^26,30^. Above 3.0 GPa, the distortion of Pb-I sublattice lowers the lattice symmetry^19,20^, which splits the degenerate scissoring and rocking bands of BA in tg and g^+^g^-^ conformations, as demonstrated by the Raman spectrum measured at 3.7 GPa. However, all Raman peaks becomes weak and disappears beyond 6.0 GPa due to the structural amorphization (Supplementary Fig. 10a). The evolution of peak position and intensity of BA scissoring and rocking modes measured by the in situ Raman spectra under compression (< 3.0 GPa) evidences the bond-by-bond rotation of the BA molecules to form distinct rotational isomers during lamellar contraction. More importantly, the irreversible changes of BA scissoring (Fig. 4d) and rocking (Fig. 4e) modes in the Raman spectra measured after decompression further proves the formation of BA tg and g^+^g^-^ conformers. Interestingly, the fingerprint of Raman bands of tg and g^+^g^-^ conformers is still clear after decompression from high-pressure amorphous state (9.8 GPa), thus the BA conformers are strain-hardened and stabilized under high-pressure treatment. At ambient conditions, the high-energy isomers could partially transform back to the tt isomer according to the moderate barrier as shown by our DFT and AIMD simulations. This reversible isomerization renders the storage of the mechanical energy and hindrance of the deformation.

In contrast, in the case of *n* = 1 RPP, previous high-pressure Raman study by us showed that the BA chain remains linear under compression, and a totally reversible Raman spectrum is obtained after decompression^21^. This implies an interesting thickness (*n*)-selective response rooted in the *n*-dependent isomerization of the BA molecule.

### Broadband photoluminescence spectra in the exfoliated RPP crystals

The changes in excitonic absorption or emission properties of bulk hybrid perovskite crystals under compression have been attributed to changes in bond lengths and bond angles of the inorganic octahedral cages, which affect the overlap of Pb 6 s and I 5p orbitals^19,31^. The exfoliated flakes are much thinner than bulk crystal and allow compression or relaxation effects to permeate much more quickly throughout the sample compared to the bulk (Supplementary Fig. 11), thus upon decompression, the elastic potential energy stored in the inorganic sublattice can be released quickly. By recording PL during compression-decompression, we can study the pressure threshold of the elastic recovery regime. In addition, surface effects are strongly amplified in the mechanical exfoliated flakes^29,32,33^, as revealed by the pronounced PL emission located at the low-energy side in addition to the intrinsic excitonic emission as shown in the Supplementary Fig. 12^22^.

**Fig. 5:**
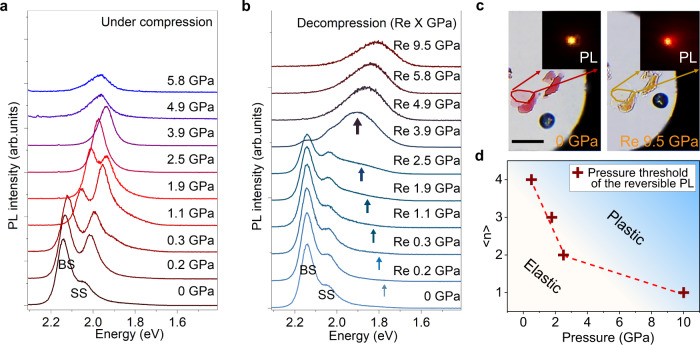
PL characterization of the reversible and irreversible deformation in RPP. *In*-*situ* PL spectra of *n* = 2 RPP; individual spectrum in (**a**) shows compression at specific “X” GPa, and the corresponding spectrum in (**b**) after decompression of “X” GPa indicated as Re “X” GPa. The blue arrows in gradient color represent the proportion of the broadband emission. **c** Optical photographs and fluorescence images of *n* = 2 RPP exfoliated flakes before and after pressure treatments. Scale bar is 50 *µ*m. **d** Plot summarizing the threshold pressure to cross from elastic to plastic regime for *n* = 1 to 4 RPPs, which is concomitant with the transition from a state where PL is reversible after decompression, to one where it is not. Dashed line guides to the eye, and the blue-shaded area represents the plastic deformation region. Source data are provided as a Source Data file.

Figure 5a shows the PL spectra of exfoliated *n* = 2 RPP flake with thickness of ~ 17 nm (~ 8 layers) that are collected under compression, while Fig. 5b shows the one-to-one corresponding spectra after decompression (refer to the Supplementary Fig. 13 for PL spectra of other two exfoliated flakes). Before compression, the sample displays a clear intrinsic excitonic emission at ~ 2.14 eV arising from the bulk-like state (BS), this corresponds to the state where the majority of its BA molecules adopt the trans configuration typical of ground state alkane molecules. A low-energy PL emission centered at ~ 2.06 eV is related to surface state (SS) emission introduced by a small population of relaxed/disordered tt-conformer BA molecules^34^. Upon compression to 2.5 GPa, the BS and SS emissions red shift continuously and merge into a single peak at 3.9 GPa (PL peak positions vs pressure shown in the Supplementary Fig. 14). This single peak blue shifts with further increase in pressures until a drastic drop in PL intensity occurs due to amorphization (> ~ 5.8 GPa), as shown in Fig. 5a and the Supplementary Fig. 14. Importantly, below the pressure of 2.5 GPa, the PL spectra can recover to the initial state after decompression, as judged from the suppression of broadband emission at the low-energy side in Fig. 5b (highlighted by blue arrow). The ability of the PL to recover is due to the elastic recovery of the Pb-I sublattice. In other words, the soft organic cation buffers the compressive strain and allows the inorganic lattice to remain in the elastic regime without crossing the yield point. At pressure higher than 2.5 GPa, e.g., 3.9 GPa, the pressure yield point is exceeded in the stress-strain curves and the PL peak broadened irreversibly. To elucidate the origins of the observed broadband PL emission, the optical absorption of the flake was recorded during compression-decompression cycles (Supplementary Fig. 15). The absorption and PL peaks exhibit similar response under compression, suggesting their excitonic origins. Both peak intensities and energies recover after pressure is released from 3.9 GPa to atmospheric pressure, while both absorption and PL peaks becomes weak and almost disappear at 3.9 GPa. Based on the above pressure-induced response, we infer that the broadband PL is associated with the radiative emission of the self-trapped excitons^35,36^. The corresponding optical photographs and fluorescence micrographs before and after compression are shown in Fig. 5c, we can observe that emission is changed from original red color to orange.

In the case of *n* = 1, the linear BA tilting along with the octahedral tilting in the out-of-plane direction works synergistically to accommodate elastic deformation, as demonstrated by the robust PL for *n* = 1 RPP even at 9.9 GPa (Supplementary Fig. 16a) and the recoverable PL after decompression (Supplementary Fig. 16b).

## Discussion

The role of the organic cations in buffering stress becomes clear when we examine the deformation resistance of the inorganic lattice for *n* = 1 and *n* = 2 RPP. As *n* increases, the ratio of the number of layers of inorganic slab (hard) to organic slab (soft) increases in the RPP homologous series from *n* = 1 to 4, and varying bands of tensile and compressive stress develop respectively. When the proportion of BA organic fraction decreases, the ability to buffer compressive stress in the hybrid system decreases. As a result, there is increasing strain in the Pb-I inorganic lattice, leading to an increasingly lower pressure threshold from *n* = 1 to 4 RPPs for permanent deformation, as demonstrated by the in situ compression-decompression PL spectra shown in the Supplementary Fig. 16. The pressure threshold is reflected by the occurrence of non-recoverable PL after decompression for *n* = 1 to 4 RPPs, as summarized in Fig. 5d. As *n* increases, the critical pressure point, above which irreversible deformation occurs, decreases, i.e., it requires a lower compressive stress to deform the inorganic lattice, as judged by the appearance of the broadband PL. This clearly corroborates the fact that the organic cation helps to buffer the stress during compression. Therefore, when the proportion of organic cations is increasingly diluted by thicker inorganic slab, the deformation resistance decreases.

In *n* = 1 RPP, the compressive stress is first accommodated by the tilting of the organic cation, and this acts in concert with the in-plane slip of the inorganic slab, which results in phase change of the crystal upon compression. The absence of methyl ammonium ions occupying the cubo-octahedral site in *n* = 1 RPP imposes less steric hindrance, and this may allow the organic chain to tilt and un-tilt in a reversible way. By contrast, the compressive stress in *n* = 2 RPPs is accommodated firstly by the rotational isomerism of the organic cations. The rotation to high energy conformer is accompanied by the in-plane tensile strain along *a* and *b* axis. Following decompression, elastic recovery of the inorganic octahedral cages in *n* > 1 can be achieved, but the pressure threshold for irreversible deformation is much lower than the *n* = 1 case. Our results provide the essential basis for understanding how multilayer organic-inorganic system act synergistically under compressive stress, which may be useful for understanding the isothermal barocaloric effects in perovskites, and for engineering new types of deformation-resistant multilayer coatings.

## Methods

### Sample preparation

The RPP single crystals were synthesized using three precursors: PbO, C_4_H_9_NH_3_I (BAI) and CH_3_NH_3_I (MAI) via a temperature-programmed crystallization method^37^. For (BA)_2_MAPb_2_I_7_ (*n* = 2), PbO, BAI, and MAI (1.9/1.4/1 by molar, MAI: 0.31 M) were dissolved in a concentrated mixture of HI and H_3_PO_2_ (9:1, v/v). The solution was then heated at 110 °C while stirring for 40 min to give clear solutions. Subsequently, the solutions were quickly transferred to an oven set at 110 °C and allowed to cool slowly down to room temperature at a rate of 3 °C h^−1^, in which the 2D RPP crystals crystallized. The crystals were isolated via vacuum filtration and subsequently dried under vacuum at room temperature^28,29^. Thin flakes of RPPs were exfoliated by following the method developed for graphene exfoliation and transferred onto a culet of 500 µm of the diamond anvil cell in the glovebox^21^.

### High pressure environment realization

Two identical diamond anvils with a culet of 500 µm were employed to generate pressure. A stainless-steel gasket was pre-indented to 50 µm with a drilled hole 300 µm in diameter serving as the sample chamber. A small ruby ball was loaded together with *n* = 2 RPP. The pressure was calibrated using the pressure-dependent ruby fluorescent technique^31^.

### Raman and PL measurements during compression-decompression cycles

Raman spectra were conducted by using Renishaw Invia Raman microscope. A solid-state 785 nm laser was used to excite Raman scattering to avoid strong PL background as well as any sample degradation. For the high-pressure PL measurements, the bulk RPP crystals were first exfoliated on polydimethylsiloxane (PDMS) following the method developed for graphene exfoliation^38^, and perovskite flakes with different thicknesses were then selected and transferred onto the pressing surface (500 µm) of the DAC using a transfer stage. The exfoliation and transfer processes were carried out in an argon-filled glove box. The RPP microsheets were covered with a silicone oil before taking out of the glove box for in situ PL measurements under compression and decompression. PL spectra were excited by 532 nm laser using WITec alpha 300RAS microscope.

### First-principles calculations

The structural evolutions under compression and decompression were simulated by using Vienna ab initio simulation package (VASP)^39^. An additional vacuum layer of thickness greater than 15 Å was adopted. The Perdew-Burke-Ernzerhof functional for the exchange-correlation potential was used together with a kinetic energy cutoff of 500 eV and a 3×3×1 Monkhorst-Pack grid. All the structures are fully relaxed until the force on each atom is less than 0.005 eV Å^−1^. The van der Waal’s interactions were treated by using DFT-D3 method. The hydrostatic model is based on the calculations and experimental results of low-dimensional materials under pressure^40–42^. More details about model construction, molecular dynamics calculations and Raman calculation can be found in the Supplementary Discussion.

## Supplementary information

Supplementary Information

## Data Availability

All data generated and analyzed in this study are included in the article and its Supplementary Information, and are also available from authors upon request. The source data is provided with this work as a Source Data file. Source data are provided with this paper.

## Source data

Source Data

## Code availability

The code supporting the findings of this study are available from the corresponding author upon request.
