# Supplementary material for: Pressure driven rotational isomerism in 2D hybrid perovskites
Source: Nat Commun. 2023 Jan 25;14:411. doi: 10.1038/s41467-023-36032-y (PMC9877019; doi:10.1038/s41467-023-36032-y)
Supplement: Supplementary file 1 — Supplementary Information [file 41467_2023_36032_MOESM1_ESM.pdf]

Supplementary Information

Pressure Driven Rotational Isomerism in 2D Hybrid Perovskites

Tingting Yin[1,†*], Hejin Yan[2,†], Ibrahim Abdelwahab[3], Yulia Lekina[1,4], Xujie Lü[5], Wenge Yang[5], Handong Sun[1], Kai Leng[6], Yongqing Cai[2*], Ze Xiang Shen[1,4*] and Kian Ping Loh[3, 6*]

[1]Division of Physics and Applied Physics, School of Physical and Mathematical Sciences, Nanyang Technological University, Singapore 637371, Singapore
[2]Joint Key Laboratory of Ministry of Education Institute of Applied Physics and Materials Engineering, University of Macau, Taipa, Macau 999078, China
[3]Department of Chemistry, National University of Singapore, Singapore 117543, Singapore
[4]Centre for Disruptive Photonic Technologies, the Photonics Institute, Nanyang Technological University, 637371, Singapore
[5]Center for High Pressure Science and Technology Advanced Research (HPSTAR), Shanghai 201203 (P. R. China)
[6]Department of Applied Physics, The Hong Kong Polytechnic University, Hung Hom, Kowloon, Hong Kong, China

†These authors contributed equally: Tingting YIN, Hejin YAN.
*Corresponding author. Email: TTYIN@ntu.edu.sg; yongqingcai@um.edu.mo; zexiang@ntu.edu.sg and chmlohkp@nus.edu.sg

Inventory of Supplementary Information:

Supplementary Figures 1-16

Supplementary Fig. 1. Simulation of the pressuring process of *n* = 2 RPP (2L) via a two-step simulation process.
Supplementary Fig. 2. Simulation of the pressure-induced compression of *n* = 1 RPP (2L).
Supplementary Fig. 3. Compression process in few-layer systems.
Supplementary Fig. 4. Top view of the layer shift for *n* = 1 and 2 RPPs.
Supplementary Fig. 5. Bond length changes under compression and decompression.
Supplementary Fig. 6. The planar projected energy surface for the BA isomers from DFT calculations.
Supplementary Fig. 7. Additional helium model and the dynamic simulated alkyl chain rotation barrier for *n* = 1 and 2 RPPs under different pressures.
Supplementary Fig. 8. The phase-space distribution and barriers of the dynamic simulated octahedral tilting in *n* = 1 RPP.
Supplementary Fig. 9. The phase-space distribution and barriers of the dynamic simulated octahedral tilting in *n* = 2 RPP.

Supplementary Fig. 10. In situ high pressure Raman spectra of n = 2 RPP.
Supplementary Fig. 11. PL evolution of n = 2 bulk RPP under compression and decompression.
Supplementary Fig. 12. Thickness-dependent PL spectra in exfoliated flakes of n = 2 RRP as a function of layer number.
Supplementary Fig. 13. In situ PL spectra of n = 2 RPP exfoliated thick flakes.
Supplementary Fig. 14. Summary of the pressure dependence of the PL peak positions for n = 2 RPP with various layer thickness.
Supplementary Fig. 15. Evolution of optical absorption spectra under compression and decompression.
Supplementary Fig. 16. Distinct PL response between n = 1 and n > 1 RPPs after pressure treatment.

Supplementary Tables1-4

Supplementary Table 1. The relative energy and transformation barrier of the BA isomers from DFT calculations.
Supplementary Table 2. Calculation by DMol of vibrational modes in the frequency region of 0-700 cm^{-1} of gas BA^+ molecule in tt, tg and g^+g^- conformations.
Supplementary Table 3. Calculation by DMol of vibrational modes in the frequency region of 700-1000 cm^{-1} of gas BA^+ molecule in tt, tg and g^+g^- conformations.
Supplementary Table 4. Comparison of frequencies of vibrational modes (700-1000 cm^{-1}) of tt- and g^+g^--BA molecules in gas form and in n = 2 RPP structure.

Supplementary Discussion

The hydrostatic pressure models, the energy landscape of BA isomers from DFT calculations and molecular dynamics (MD), MD calculations for n = 1 and n = 2 RPPs, and Raman intensity calculations.

Supplementary References

Supplementary Figures

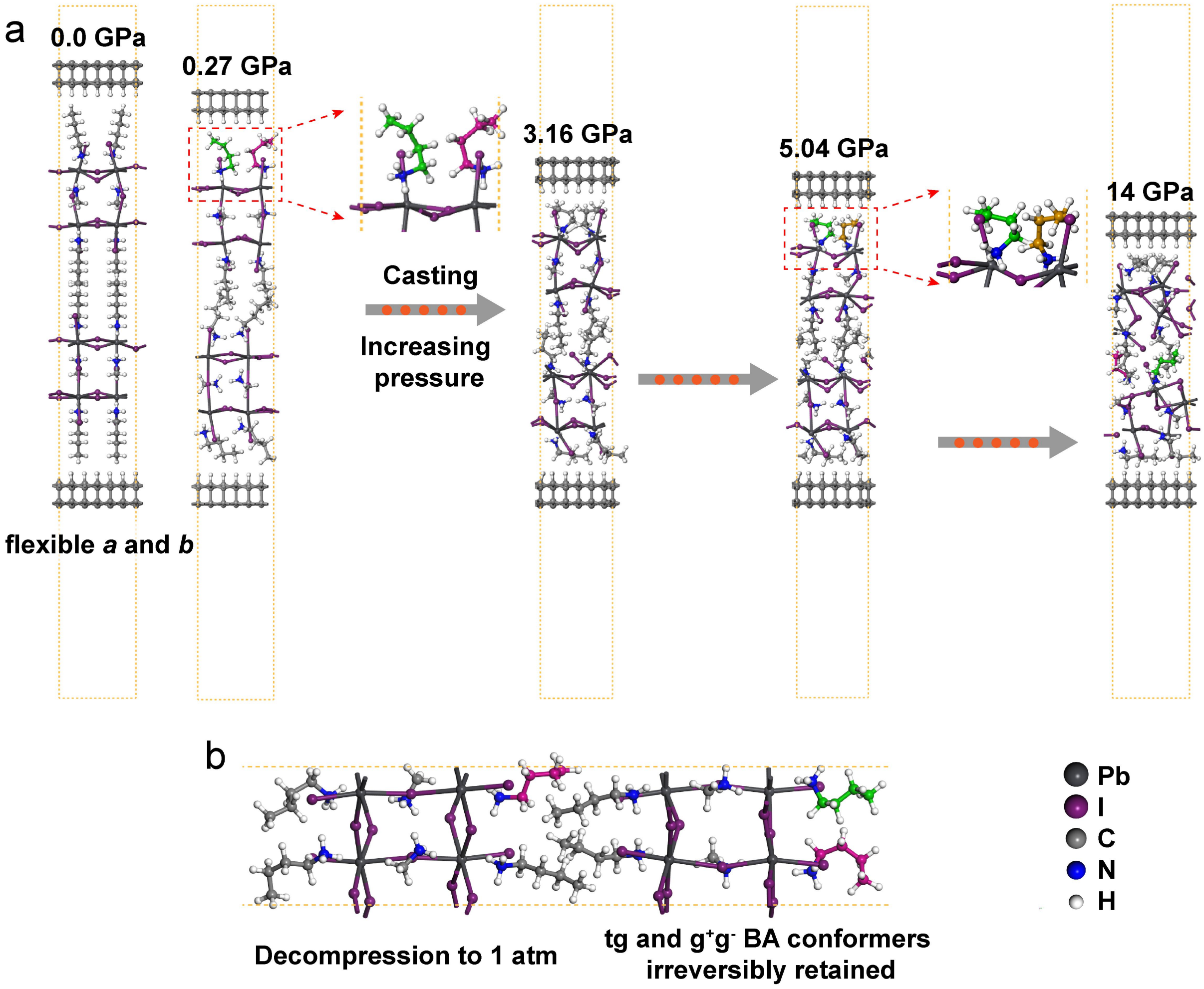

Supplementary Figure 1. Simulation of the pressurization process of *n* = 2 RPP (2L) via a two-step simulation process. a The pressure-induced lamellar contraction and BA isomerization of *n* = 2 RPP. **b** Decompressed *n* = 2 RPP structure with retained BA isomers.

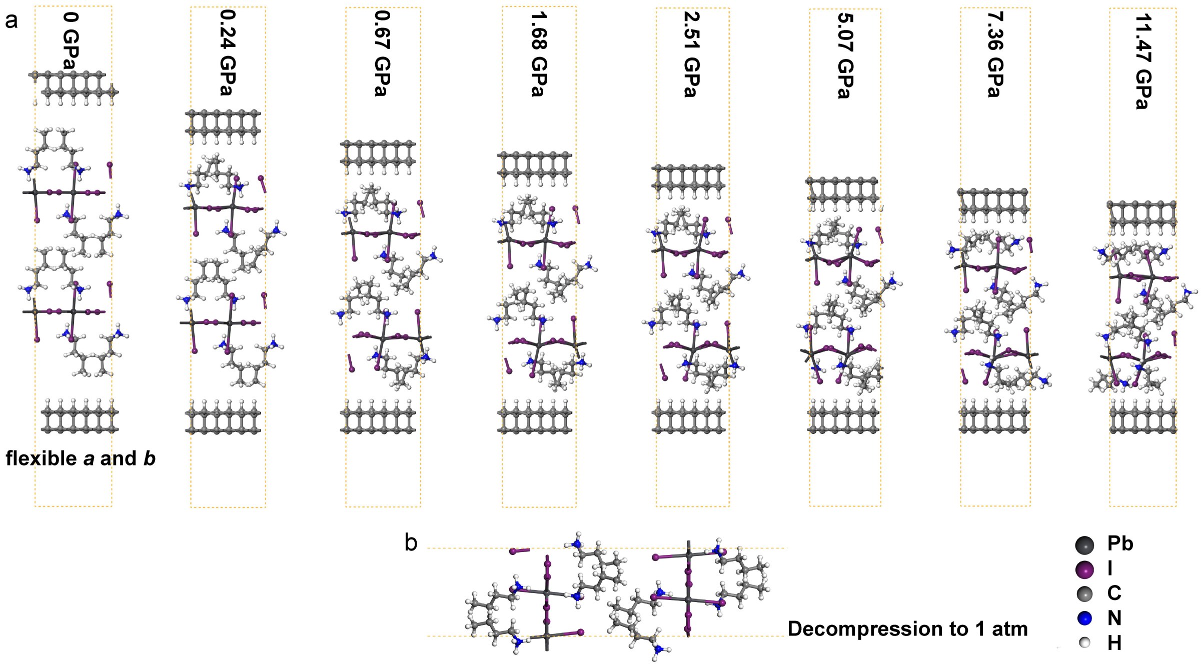

Supplementary Figure 2. Simulation of the pressure-induced compression of *n* = 1 RPP (2L). a The pressure-induced lamellar contraction and BA tilting of *n* = 1 RPP. **b** Decompressed *n* = 1 RPP structure with untilted BA isomers.

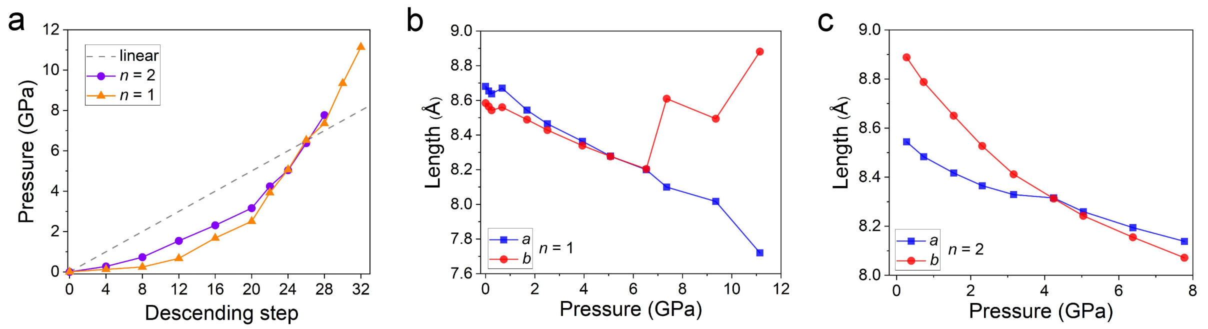

Supplementary Figure 3. Compression process in few-layer systems. a The values of the pressure in simulation were determined by stepwise shift of diamond layer descending in an interval of 0.4 Å. The dashed line represents a normal linear pressure-loading process for guiding to the eye. **b** and **c** show the lattice parameters of the bulk phase *n* = 1 and 2 RPPs at corresponding hydrostatic pressure values determined in (**a**). A clear phase transition occurs in the *n* = 1 bulk phase RPP from the orthorhombic to monoclinic at ~ 5 GPa[1]. No phase transition happens in the *n* = 2 bulk phase RPP.

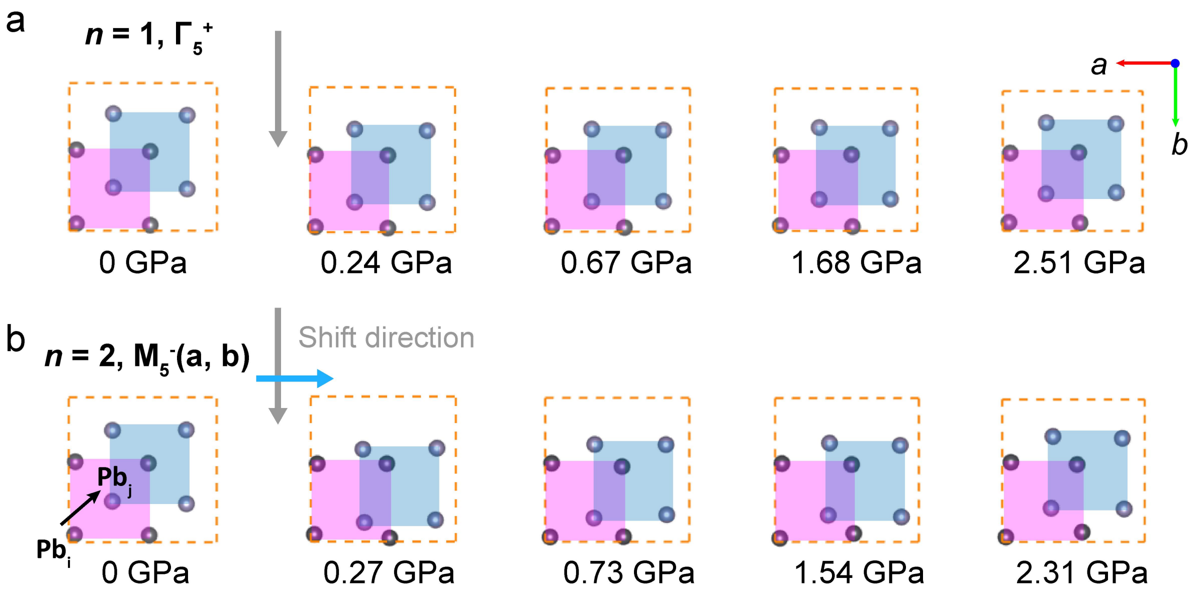

Supplementary Figure 4. Top view of the layer shift for *n* = 1 and 2 RPPs. a In *n* = 1 RPP, the layer-to-layer shift is along *b* direction related to Γ_5^+ symmetry mode. **b** In *n* = 2 RPP, the layer-to-layer shift is along both - *a* and *b* directions equivalent to M_5^- symmetry mode. Top layer of RPP is shaded in blue and bottom layer of RPP is shaded in pink.

The $\sqrt{2} \times \sqrt{2}$ supercell metrics are used to maintain the octahedral unit. The *LSF* value changes within (0, 0.5) range, its minimum value 0 and maximum value 0.5 corresponding to the standard Dion-Jacobson (DJ) and RP perovskite structure, respectively.

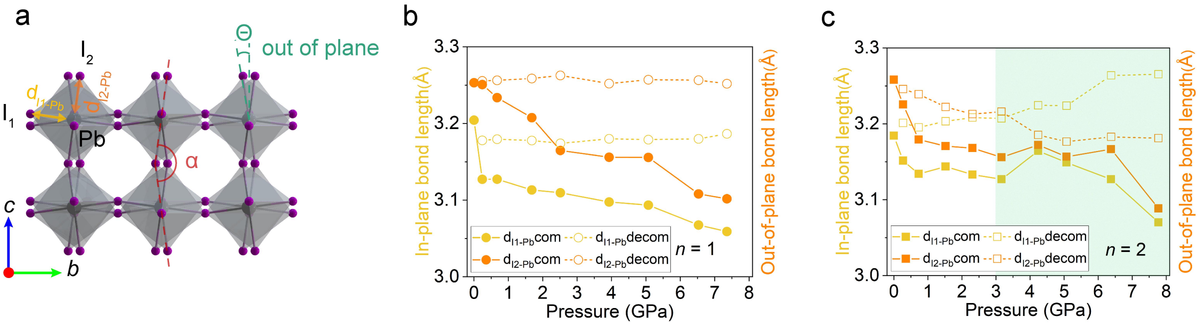

Supplementary Figure 5. Bond length changes under compression and decompression. a Schematic of the *n* = 2 RPP 2D Pb-I octahedral cages. The evolution of in-plane and out-of-plane bond lengths of *n* = 1 RPP (**b**) and *n* = 2 RPP (**c**) under compression and decompression. The green-shaded area in (**c**) represents the irreversible deformation under high pressure.

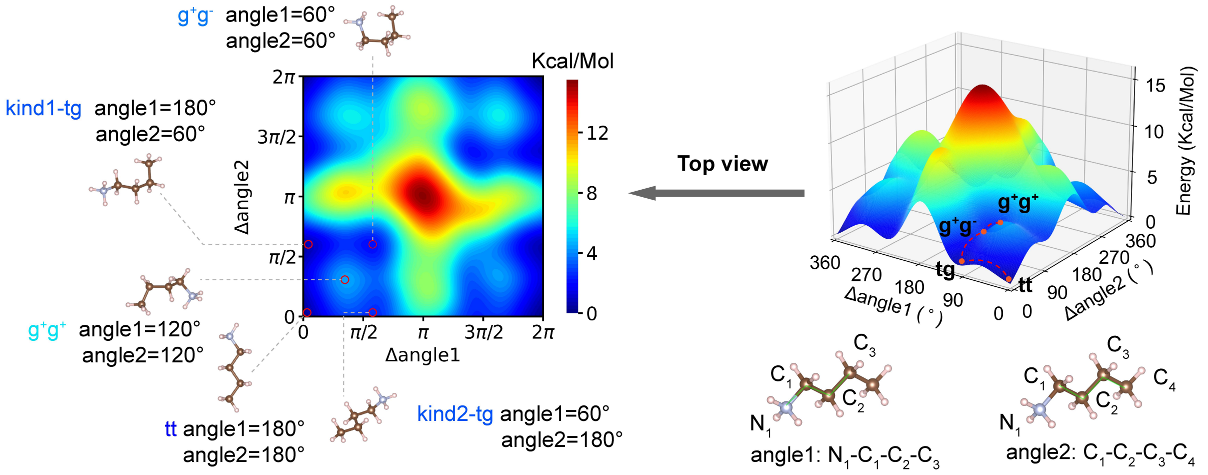

Supplementary Figure 6. The planar projected energy surface for the BA isomers from DFT calculations. Th global energy minimum tt configuration is set to zero energy with the schematics for dihedral angle1 and angle2 listed on the right side.

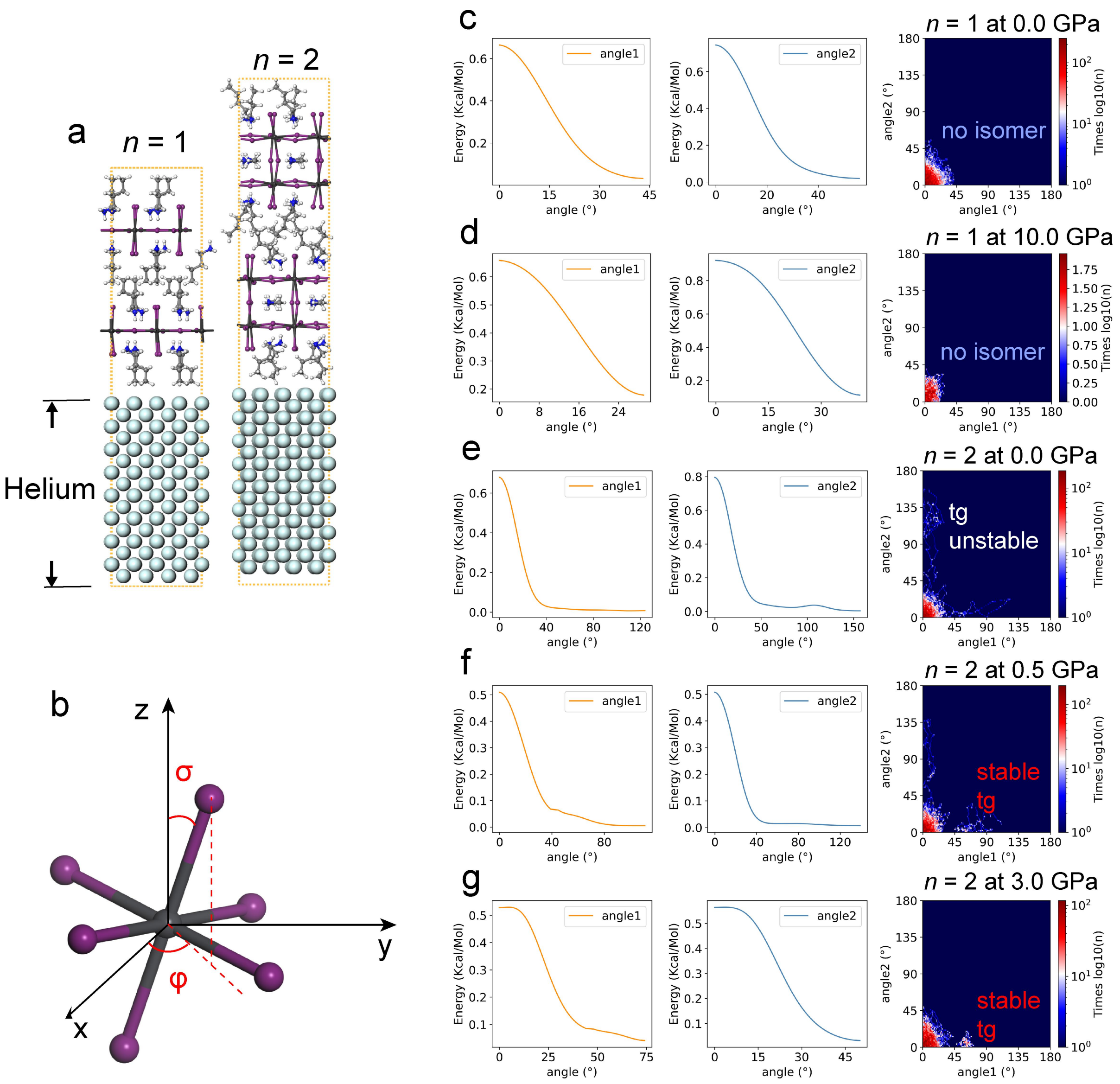

Supplementary Figure 7. Additional helium model and the dynamic simulated alkyl chain rotation barrier for *n* = 1 and 2 RPPs under different pressures. a The inert helium serves as a medium to conduct force with the three cell lattices being flexible. **b** The definition of octahedral tilting angles σ and φ for the internal coordinate of an isolated octahedral unit. The alkyl chain rotation barrier for *n* = 1 RPP (**c, d**) and *n* = 2 RPP (**e-g**) with the conformal change angle1 and 2 of BA isomers. The color bar indicated the frequency of the BA molecule at a certain configuration. No new isomers occur in the *n* = 1 RPP within 10 GPa. The critical pressure is lowered to be 0.5 GPa for the *n* = 2 RPP as the newly formed stable tg isomers.

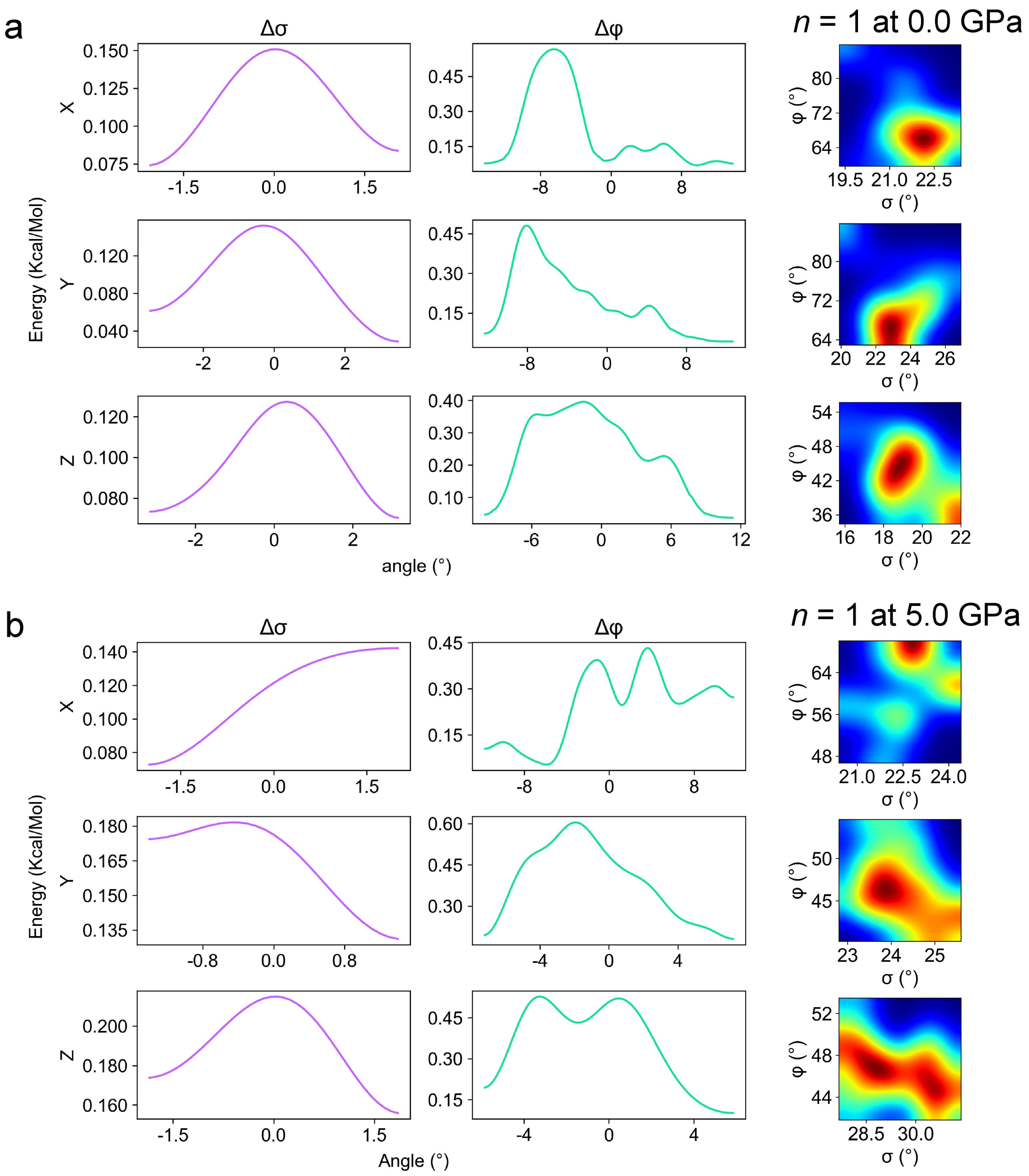

Supplementary Figure 8. The phase-space distribution and barriers of the dynamic simulated octahedral tilting in *n* = 1 RPP. The angle φ and angle σ of *n* = 1 RPP calculated at 0 GPa (**a**) and at 5.0 GPa (**b**).

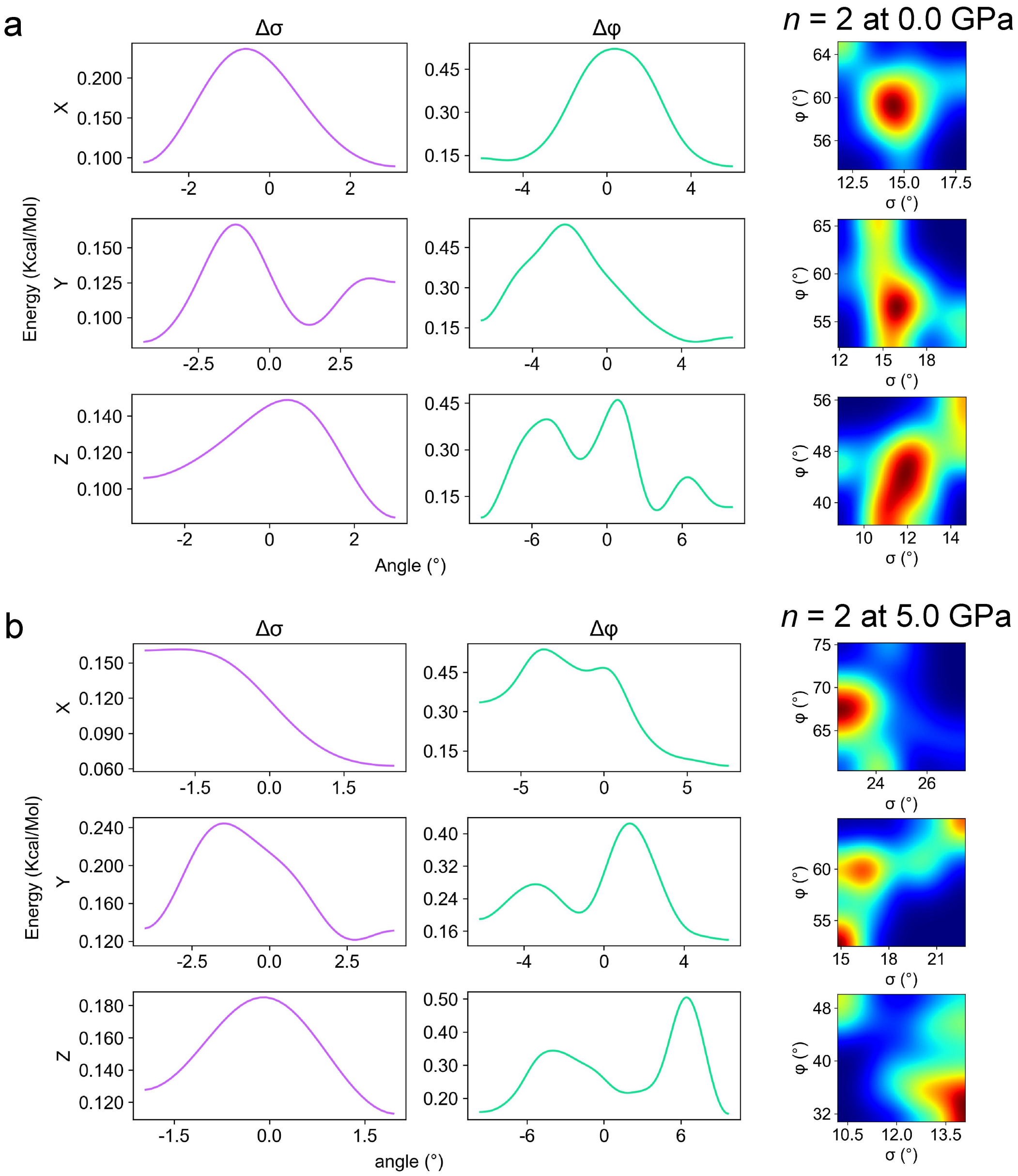

Supplementary Figure 9. The phase-space distribution and barriers of the dynamic simulated octahedral tilting in *n* = 2 RPP. The angle φ and angle σ of *n* = 2 RPP calculated at 0 GPa (**a**) and at 5.0 GPa (**b**).

The Pb-I skeleton shows strong anharmonicity within the dynamic tilting process, which can be linked to the stereochemical expression of the lone pair electrons in Pb atoms.[2, 3] As shown in Fig. S8 and S9, the angle φ in MD has a double wall potential with a height of ~ 0.25-0.60 Kcal/Mol, and the angle σ in MD is restricted in a smaller region with a shallow wall at a height of ~ 0.05-0.25 Kcal/Mol.

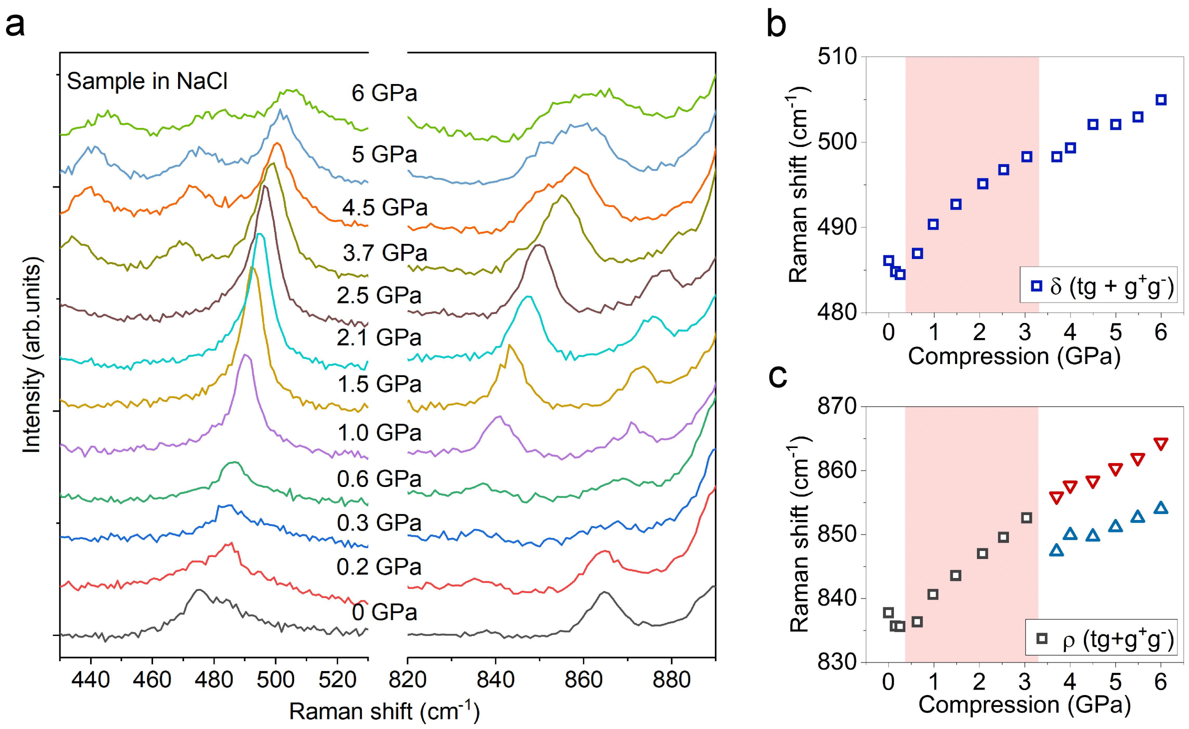

Supplementary Figure 10. In situ high pressure Raman spectra of *n* = 2 RPP. a The evolution of scissoring and rocking modes of BA under compression. **b** and **c** Raman peak position as a function of pressure for the scissoring and rocking modes.

The red shaded area (0.6-3.0 GPa) in b and c shows a continuous blue shift for both modes, reflecting contraction of the layer-to-layer distance with an increasing in pressure and the stability of the formed BA conformers. The splitting of Raman peak of the rocking mode and the discontinuity of Raman peak of the scissoring mode between 3.0 and 3.7 GPa should be due to the lowered inorganic lattice symmetry at the higher pressures.

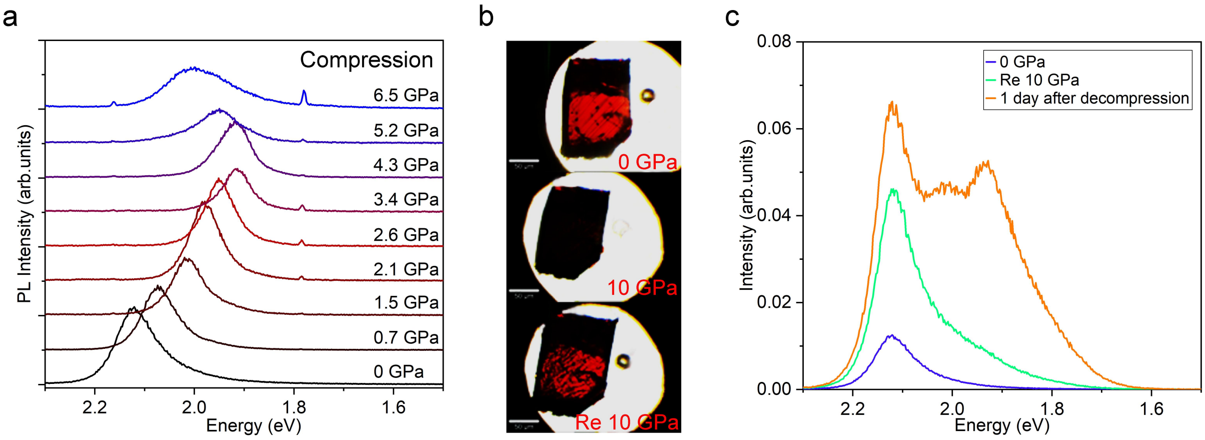

Supplementary Figure 11. PL evolution of *n* = 2 bulk RPP under compression and decompression. a In situ PL spectra of *n* = 2 bulk RPP. **b** Optical photographs of *n* = 2 bulk RPP before and after pressure treatments. The scale bar is 50 μm. **c**

PL spectra of n = 2 bulk RPP after pressure released back to 1 atm. The broadband emission is delayed for the bulk crystal sample.

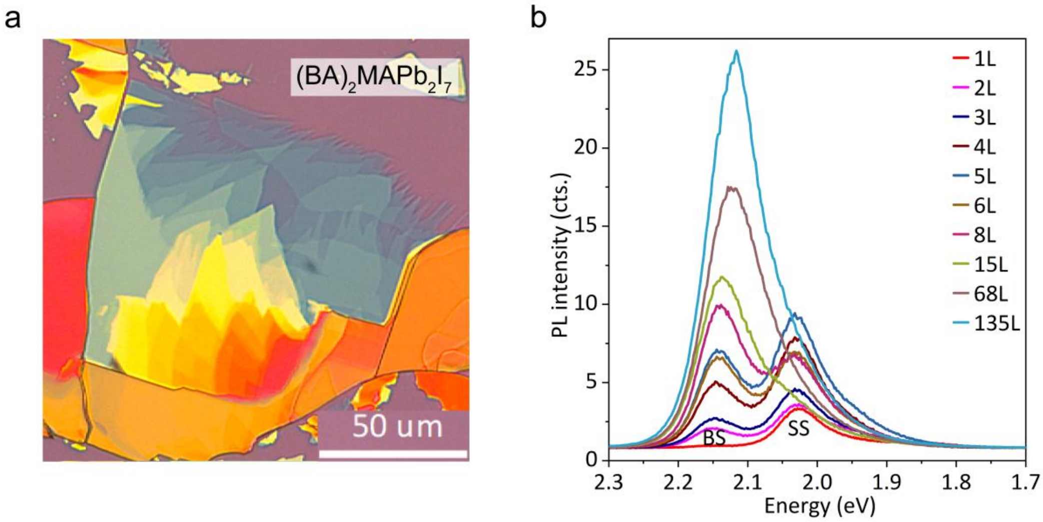

Supplementary Figure 12. Thickness-dependent PL spectra in exfoliated flakes of *n* = 2 RRP as a function of layer number. a Optical image of exfoliated flakes on Si/SiO_2 substrate. **b** Layer-dependent PL spectra. Besides the intrinsic excitonic emission at ~ 2.12-2.16 eV, the thickness-dependent PL results demonstrate a clear additional emission at low energy region of ~2.02-2.05 eV, which becomes a clear emission peak in ultrathin layer samples and contributes to the asymmetric PL lineshape for thick samples. This addition emission peak is originated from the surface states (SSs), which are introduced by the disordered tt-BA molecules on the surface of the exfoliated flakes. Thus, we can define these two peaks as bulk states (BSs) and surface states (SSs). The thickness of the mechanical exfoliated flakes is determined by from both AFM measurements and optical contrast.[4]

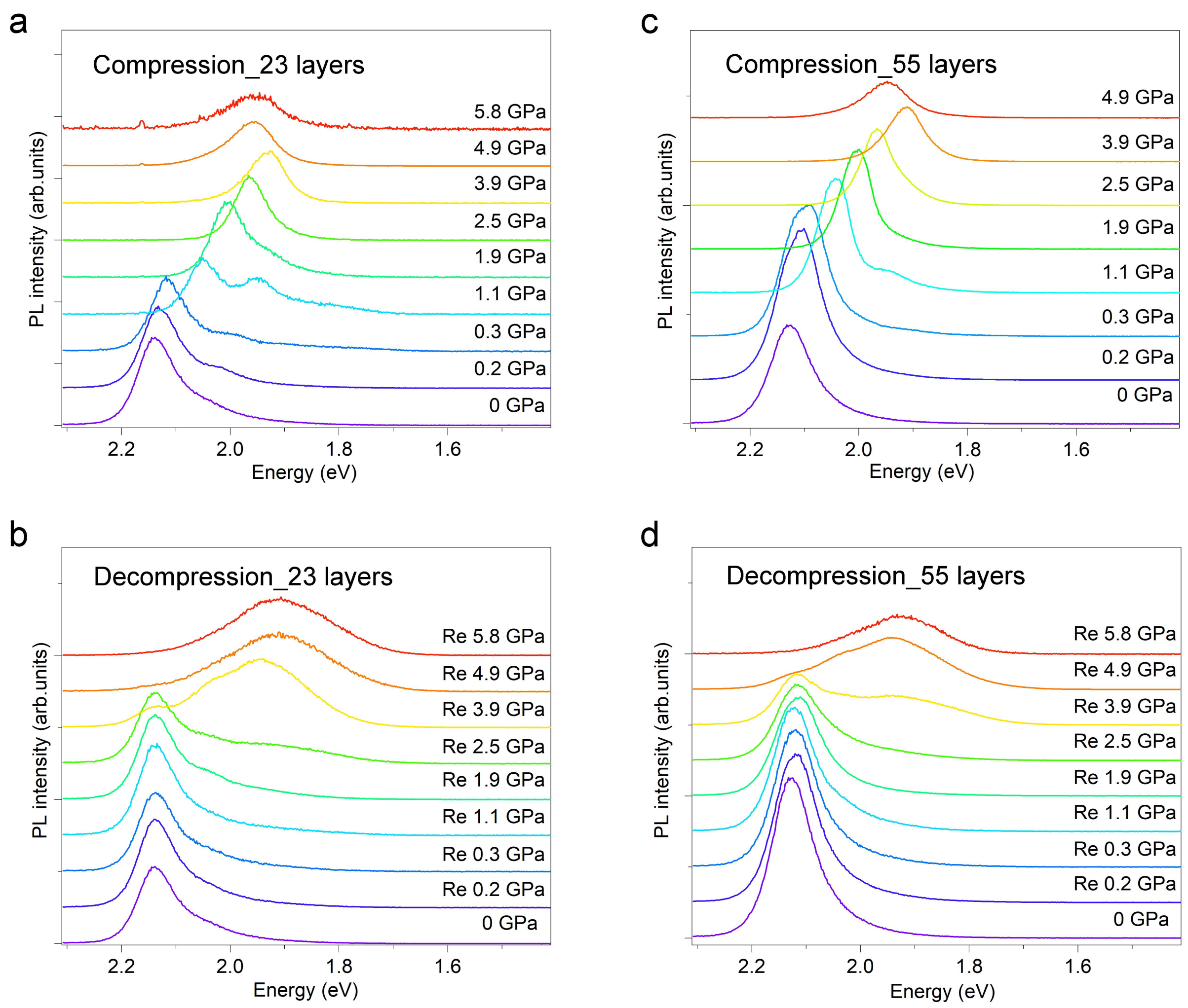

Supplementary Figure 13. In situ PL spectra of *n* = 2 RPP exfoliated thick flakes. PL spectra of 23-layer flakes (a, b) and 55-layer flakes (c, d) under compression and after one-to-one decompression.

Such broadband emission is also observed immediately in other thick flakes after pressure released from certain pressure points similar to that observed in 8-layer sample shown in the main manuscript.

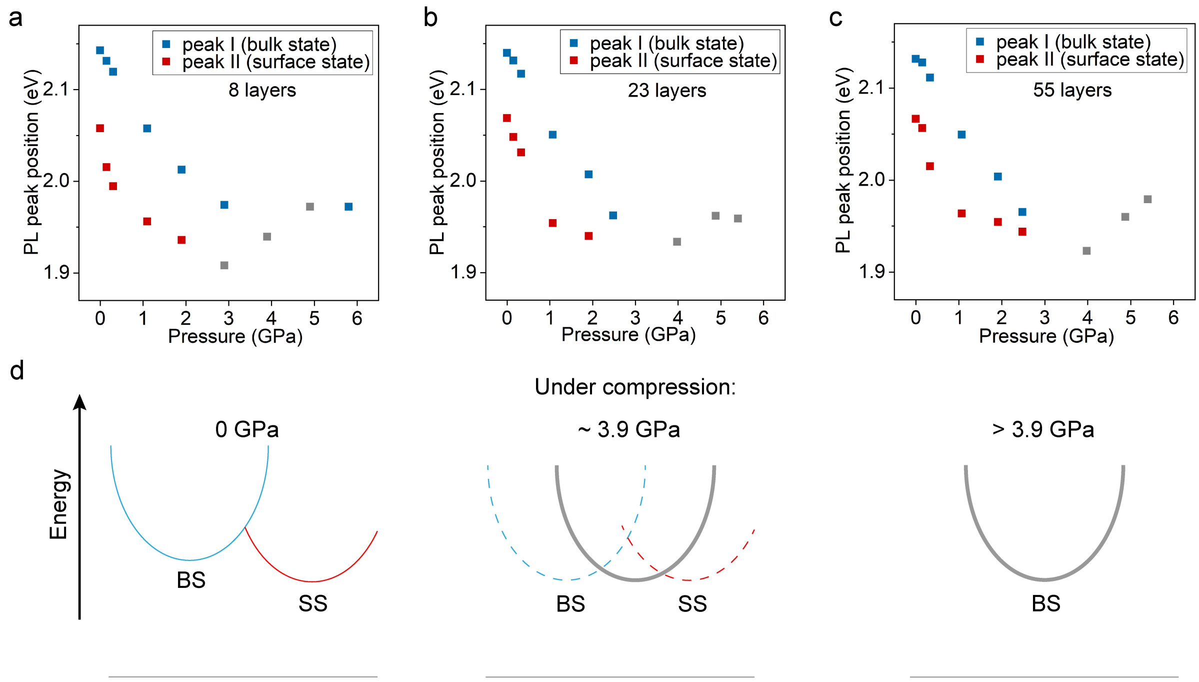

Supplementary Figure 14. Summary of the pressure dependence of the PL peak positions for *n* = 2 RPP with various layer thickness: 8 layers (**a**), 23 layers (**b**) and 55 layers (**c**). **d** Illustration of the energetic variations of BS and SS during compression. The blue band is the intrinsic BS and the red band is the intrinsic SS.

Under compression, both distinct pressure response between BS and SS with increasing pressure (< 3.9 GPa), resulting in the band merging of them and contributing to the one peak emission in the measured PL spectra in Fig. 5a in the main manuscript. Since organic layer compression is dominant in the low-pressure range, such band evolution between BS and SS should be related to the formation of BA conformers within slightly distorted Pb-I lattices. Beyond 3.9 GPa, the severe distorted Pb-I inorganic lattices should result in the blue shift of this merged state.

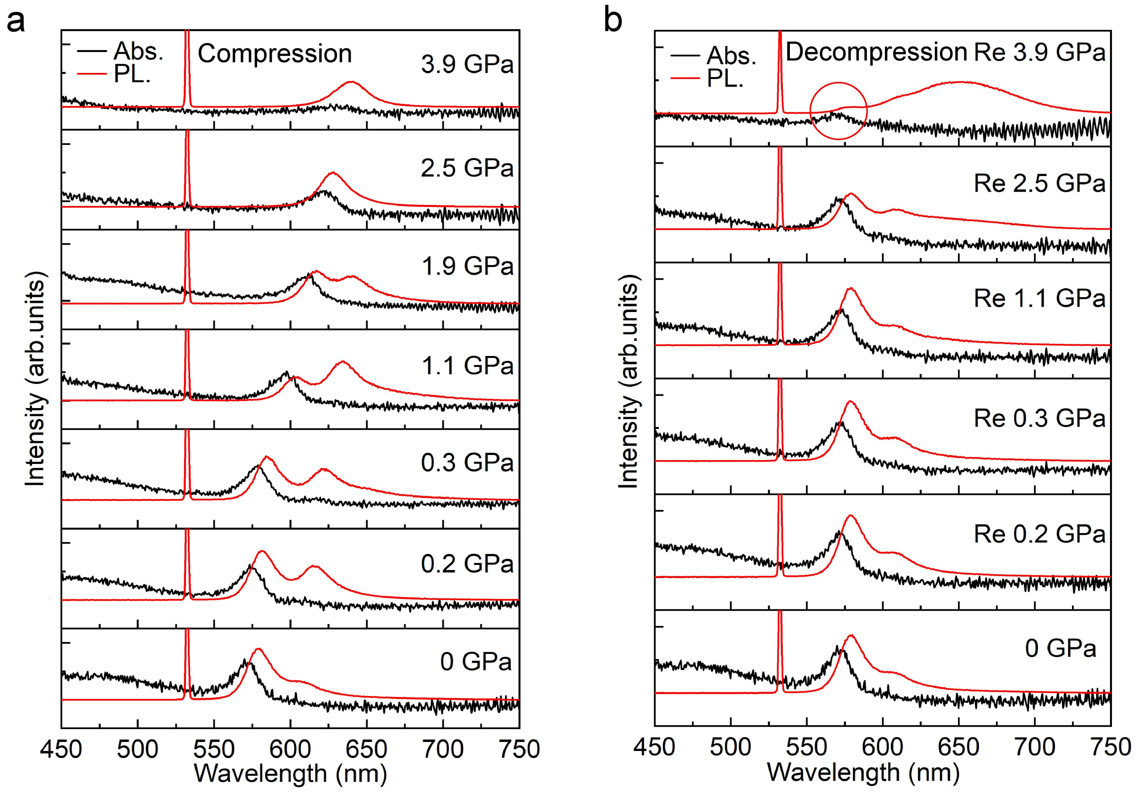

Supplementary Figure 15. Evolution of optical absorption spectra under compression and decompression. In situ optical absorption spectra (the black lines) of n = 2 RPP exfoliated 8-layer flakes under compression (**a**) and after one-to-one decompression (**b**). The corresponding PL spectra (the red lines) are overlaid here for easy comparison.

The exciton absorption peak is obtained by using exfoliated 8-layer flakes. The absorption peak of excitonic band exhibits similar pressure response to that of PL spectra, which evidences that the PL peaks are excitonic in nature. The disappearing of absorption is due to the severe inorganic lattice distortion. More importantly, the excitonic band absorption is recovered when pressure is completely released back to 1 atm from pressure point below 3.9 GPa, accompanying with clear excitonic emission in the PL spectra in the decompressed sample. While the broadband PL occurs along with the almost disappearance of excitonic absorption signal at 3.9 GPa. Thus, it demonstrates that such broadband PL emission is from the self-trapped excitons due to pressure-induced plastic deformation of inorganic lattice in n = 2 RPP.

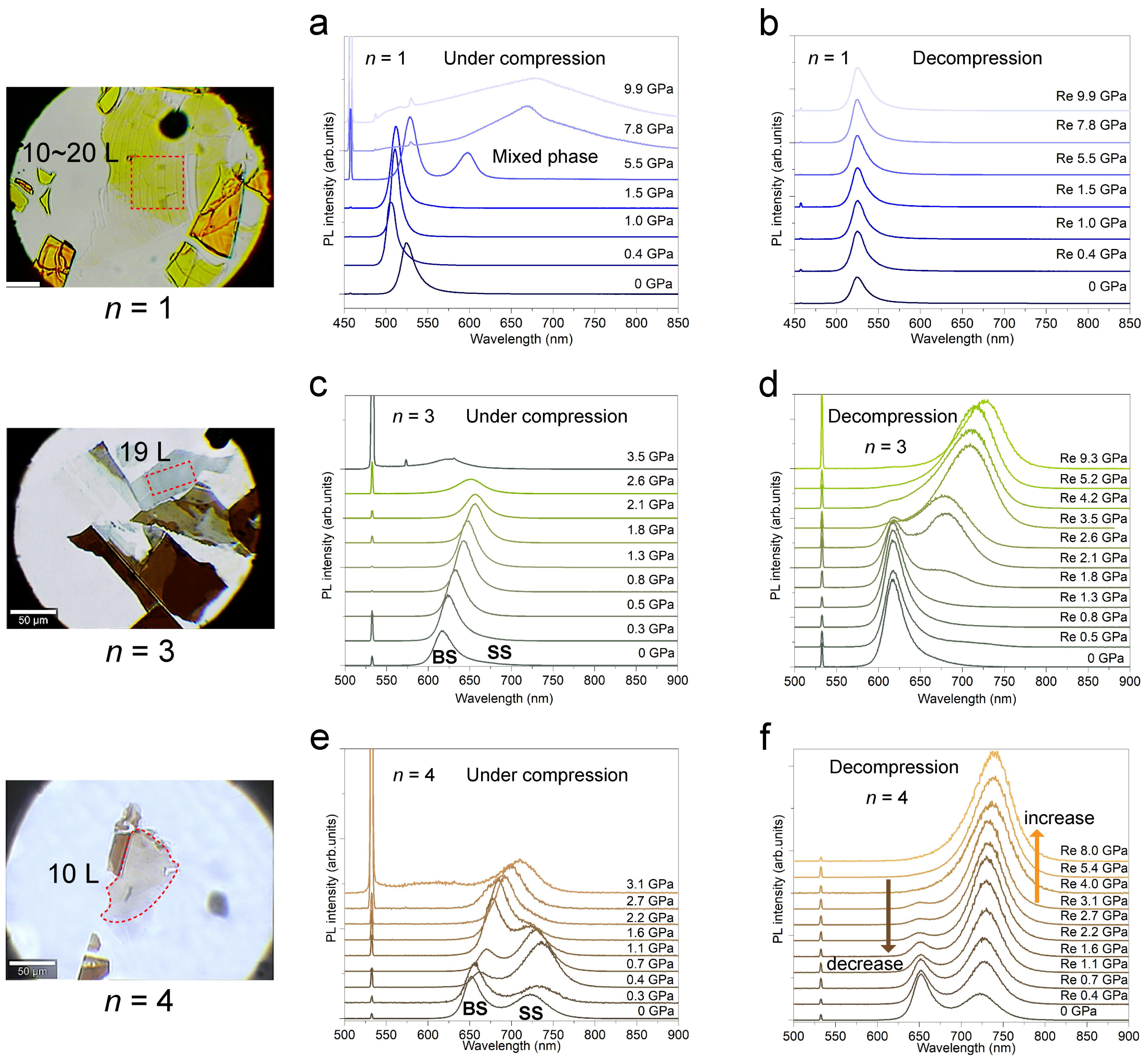

Supplementary Figure 16. Distinct PL response between *n* = 1 and *n* > 1 RPPs after pressure treatment. In situ PL spectra of *n* = 1 (a, b), *n* = 3 (c, d) and *n* = 4 (e, f) RPP exfoliated thin flakes under compression and after one-to-one decompression. The corresponding photographs of *n* = 1, 3 and 4 RPP exfoliated flakes in the DAC taken at 0 GPa are shown in the left column. The scale bar is 50 μm. The excitation wavelength for *n* = 1 RPP is 457 nm and 532 nm for *n* = 3 and 4 RPPs.

Supplementary Tables

Supplementary Table 1. The relative energy and transformation barrier of the BA isomers from DFT calculations.

Chain isomers	Energy (Kcal/Mol)	Barrier (Kcal/Mol)
BA in tt form	0	0
BA in tg form	0.6-1.6	1.1-2.8

BA in g^+g^- form	2.1	3.2
BA in g^+g^+ form	4.5	4.5

Supplementary Table 2. Calculation by DMol of vibrational modes in the frequency region of 0-700 cm^{-1} of gas BA$^+$ molecule in tt, tg and g^+g^- conformations. We have obtained seven Raman active modes for BA vibrations in the frequency range of 0-700 cm^{-1}. Mode 5 shows the highest Raman intensity for the tt-BA molecular vibrations, mode 6 and 7 have the highest Raman intensity in the tg arrangement, and mode 7 has the highest Raman intensity in the g^+g^- arrangement.

BA^{+1} modes	tt-BA frequency	tt-BA intensity	tg-kind1 BA frequency	tg-kind1 BA intensity	tg-kind2 BA frequency	tg-kind2 BA intensity	g^+g^--BA frequency	g^+g^--BA intensity
Mode 1	134.4	2.16×10^{-5}	105.63	1.91×10^{-4}	96.17	1.07×10^{-4}	47.26	6.85×10^{-4}
Mode 2	186.68	2.40×10^{-4}	120.57	2.30×10^{-4}	139.72	3.54×10^{-4}	179.17	2.89×10^{-5}
Mode 3	258.8	3.43×10^{-5}	212.56	6.49×10^{-4}	198.54	2.51×10^{-5}	252.71	3.58×10^{-5}
Mode 4	356.66	7.47×10^{-5}	278.3	1.92×10^{-4}	254.63	4.77×10^{-5}	310.27	1.34×10^{-4}
Mode 5	404.20	1.29×10^{-3}	331.92	4.65×10^{-4}	299.89	1.11×10^{-4}	352.62	1.36×10^{-4}
Mode 6			475.79	3.37×10^{-4}	337.96	2.06×10^{-4}	387.79	4.57×10^{-5}
Mode 7					491.75	3.55×10^{-4}	481.30	7.41×10^{-5}

Supplementary Table 3. Calculation by DMol of vibrational modes in the frequency region of 700-1000 cm^{-1} of gas BA$^+$ molecule in tt, tg and g^+g^- conformations. We have obtained six Raman active modes for BA vibrations in the frequency range of 700-1000 cm^{-1}. Mode 2 has the highest Raman intensity for tt-BA, two mode 3 are Raman active in the tg-BA, and mode 1 is active in g^+g^--BA. However, the frequency of mode 2 is redshifted a lot from 863.57 cm^{-1} in the tt conformation to 863.11(836.62) and 777.61 cm^{-1} in the tg and g^+g^- arrangements.

BA^{+1} modes	tt-BA frequency	tt-BA intensity	tg-kind1 BA frequency	tg-kind1 BA intensity	tg-kind2 BA frequency	tg-kind2 BA intensity	g^+g^--BA frequency	g^+g^--BA intensity
Mode 1	804.70	1.66×10^{-5}	760.24	1.28×10^{-4}	759.37	5.26×10^{-5}	777.61	4.68×10^{-4}
Mode 2	863.57	1.09×10^{-3}	813.27	1.59×10^{-4}	818.19	8.21×10^{-4}	820.11	1.28×10^{-4}
Mode 3	907.59	2.84×10^{-4}	863.11	1.06×10^{-3}	836.62	1.86×10^{-3}	882.17	1.37×10^{-5}

Mode 4	984.40	1.46×10^{-4}	889.60	1.29×10^{-4}	904.24	3.31×10^{-5}	904.99	7.22×10^{-5}
Mode 5			918.40	1.21×10^{-5}	927.15	2.59×10^{-4}	983.93	1.11×10^{-4}
Mode 6			971.61	2.91×10^{-4}	985.77	4.98×10^{-5}		

Supplementary Table 4. Comparison of frequencies of vibrational modes (700-1000 cm^{-1}) of tt- and g$^+$g$^-$-BA molecules in gas form and in *n* = 2 RPP structure. To test the effect of PbI_4^{2-} inorganic lattice on the BA vibrational evolution from tt to g$^+$g$^-$ conformation, we perform similar phonon frequency calculations by VASP package. Mode 2 still shows a red-shift trend from the tt to g$^+$g$^-$ conformational change even in *n* = 2 RPP structure.

BA^{+1} modes	Gas form (tt)	Gas form (g$^+$g$^-$)	In RPP (n=2) (tt)	In RPP (n=2) (g$^+$g$^-$)
Mode 1	804.70	777.61	790.2	752.1
Mode 2	863.57	820.11	890.5	803.5
Mode 3	907.59	882.17	894.1	760.7
Mode 4	984.40	904.99	953.4	891
Mode 5		983.93		938.5

Supplementary Discussion

The hydrostatic pressure models:

To simulate the hydrostatic pressure-driven isomerization process in the few-layer samples, atomic models and lattice were relaxed by DFT simulations for all three directions as schematically shown in Fig. S1. A two-step approach was adopted to reproduce the shrinkage of the vdW gap and the in-plane lattice. Firstly, top and bottom hydrogenated diamond plates and pressure exerted along out-of-plane (*c*) direction by consecutive downward shift with their atomic positions fixed during relaxation. The diamond layers also serve as pressure gauges by summation of the residual atomic forces along the *c* direction of each top/bottom layer of nanodiamond. The stepwise, linear movement along the *c* direction thus generates a series of pressure values following a nonlinear power-law trend rather than the linear pressure interval in the experiment as shown in Fig. S2a. In the second step, a hydrostatic simulation was conducted for the bulk RPP phase under above pressure values. The lattice parameters of the *a* and *b* directions in few-layer RPPs are then determined by the lattice constants of their corresponding pressurized bulk RPP phase. The evolutions of the *a* and *b* lattice parameters as pressure are represented in Fig. S2b and c.

The energy landscape of BA isomers from DFT calculations and molecular dynamics (MD):

DFT calculations were performed to examine the relative energy landscape of the free BA molecule in vacuum within the phase space of two backbone angles less than 2π radian. The conformational notation convention, e.g., t = trans (180°) and g = gauche (± 60°), labels these isomers, additional ± superscripts indicate two angles at the same or opposite sign. The tt configuration is the global minimum state which is set as the reference state with the zero of energy, while tg and g^+g^- are two local minimum states with a relative energy of ~ 0.6-1.6 Kcal/Mol and ~ 2.3 Kcal/Mol above the tt ground state. The g^+g^+ is a local maximum state with relative energy of ~ 4.5 Kcal/Mol. This energy surface also indicates that the transitional pathway from tt to tg and tt to g^+g^- with the saddle-like barriers of ~ 1.1-2.8 and 3.2 Kcal/Mol, respectively.

MD calculations were performed to consider the temperature contribution and the organic-inorganic interactions of RPPs (The calculation details are listed in the next section.). The inner molecular rotation barrier for BA cation within RPP layers are reduced to ~ 0.5-0.9 Kcal/Mol at 300 K, thus the atomic thermal vibrations reduce barrier height as compared to that of ~ 0.6-2.3 Kcal/Mol obtained by DFT calculations at 0 K, which should be due to the atomic thermal vibrations. Notably, the pressure has opposite contribution to the barrier of BA isomerization in *n* = 1 and 2 RPPs. Pressure promotes the inner molecular rotation in *n* = 2 RPP and lowers the energy barriers of ~12.5-25% at 3 GPa, while pressure restricts the molecular rotation in *n* = 1 RPP. Besides, the energy barrier for octahedral tilting is relatively smaller than that of isomerization. The inorganic layer has certain degree to absorb the pressure by octahedral tilting before, during and after isomerization.

MD calculations for *n* = 1 and *n* = 2 RPPs:
To investigate the temperature effect on the alkyl cation isomerization and inorganic octahedral tilting, we performed additional examination based on a helium pressure-medium model which allows a straightforward simulation of hydrostatic deformation via cell relaxation. Similar to the nanodiamond, the helium layer acts as the pressure medium. Since the thickness of adopted helium layer is 16 atomic layers (~ 25 Å), such setting ensures the thickness of the helium remains above 10 Å even under high pressure of 12 GPa and eliminates the impact from image atoms. This helium medium model has been successfully applied to simulate low-dimensional materials under hydrostatic pressure.[5] The *ab initio* molecular dynamics simulations (AIMD) were performed through the Quickstep suite in the CP2K package.[6] Based on the prototype cubic phase lead halide perovskites, similar calculation parameters were adopted here.[7, 8] The GGA of the truncated PBE0 (trPBE0) functions, including 25% of Hartree-Fock exchange, were used to treat the electronic exchange-correlation energy. The embedded auxiliay density matrix method in the CP2K allows these expensive hybrid calculations both feasible and applicable. The double-zeta quality basis sets (DZVP-MOLOPT) and Goedecker-Teter-Hutter (GTH) pseudopotentials were chosen combined with the dispersion corrections at the Grimme's DFT-D3 level without damping. The cutoff energies were set to be 500 Ry and 50 Ry for the plane-

wave bases and PBE0 functions, respectively. The enlarged $\sqrt{2} \times \sqrt{2} \times 1$ supercells of the layered n = 1 and n = 2 RPPs were adopted for the dynamic compression under certain external pressure. The energy minimizations were performed before dynamics calculations. Equilibrium dynamics were achieved in the NPT_F ensemble which allows the flexible supercell size and shape. Each dynamics calculation was performed with a time length of 5 ps at a time step of 0.5 fs. Considering the dynamic tilting of the inorganic octahedral units, a pair of azimuthal angles (σ, φ) and corresponding probability distribution p(σ, φ) can be used to determine the entropic contributed tilting energies as[8]:

$$F(\sigma_i) =- k_B T\, In(\int d\varphi_i\, p(\sigma_i), \varphi_i), \quad (1)$$

$$F(\varphi_i) =- k_B T\, In(\int d\sigma_i\, p(\varphi_i), \sigma_i). \quad (2)$$

where the σ_i and φ_i is the azimuthal angles for a certain axis-i (i=x, y, z). Similar method can be extended to calculate the entropic contributed rotational barriers of BA isomers of the backbone angle1 (N_1-C_1-C_2-C_3) and angle2 (C_1-C_2-C_3-C_4) within the dynamic process.

The Raman intensity calculation:
Raman intensities for BA molecules in gas form were calculated by using the DMol package.[9] The frequency of the molecular intra-molecular vibrational modes tends to be affected by the inorganic lattice within the molecule-lattice crystalline field. Due to the massive computational resources needed for the calculation of the Raman spectra of the 1L n = 2 RPP, we cannot provide the intensity of the Raman modes of the BA molecule embedded in the inorganic lattice. To include the effect from inorganic lattice, the frequency of the Raman mode of BA molecules were performed in the n = 2 RPP structure by using VASP package. Besides, to compare the vibrational frequencies of the Raman modes for the BA molecule in gas form and in n = 2 RPP phase, we performed DFT calculations via displacement method using the efficient VASP package.[9] The LDA function and a 3x3x1 k-grid were used in the simulation. To identify the phonon modes involved with the BA molecule, we analyzed the phonon eigenvector and calculated the relative weight of the vibrational amplitudes of the BA atomic groups over the total atoms. The renormalization of the phonon frequencies of Raman mode of the tt and g^+g^- BA molecules with and without the effect of Pb-I lattice can thus be obtained.

Fig 4a and b correspond to the theoretically simulated Raman spectra for the BA scissoring and rocking modes in the tt, tg-kind1 (tg-kind2) and g^+g^- structures of the BA gas molecules. The calculated Raman peak in the low-frequency region between 350 - 550 cm^{-1} (the dark grey line in Fig. 4a) is from the superposition of two skeletal scissoring modes of BA in the trans form, which belong to scissoring vibrations of CH_2-CH_2-CH_3 (δ(CCC)) and CH_2-CH_2-NH_3^+ (δ(CCN)), respectively (the vibrational patterns are inserted in Figure 4c). This mode is hardened after evolving into the BA conformers, *i.e.*, tg (~ 475.79 (491.75) cm^{-1}, dark and light blue lines) and g^+g^- (~ 481.3 cm^{-1}, red line), as demonstrated by the vibrational patterns in Fig. 4a. On the

other hand, the calculated Raman peak in the high frequency region of 730 - 950 cm^{-1} (the dark grey line in Fig. 4b) is assigned to the skeletal rocking modes of BA in the trans form, *i.e.*, (ρ(tt-BA)), which have a strong rocking (CH_3, NH_3^+) contribution but is coupled with the CH_2-CH_2-CH_2 stretching (the vibrational pattern is inserted in Figure 4c). This mode at 863.57 cm^{-1} becomes weak while Raman modes at ~ 863.11 (818.19) cm^{-1} and 777.61 cm^{-1} appear after evolving into the ρ(tg-BA) (dark and light blue lines) and the ρ(g^+g^--BA) (red line), as demonstrated by the vibrational patterns in Fig. 4b.

Supplementary References

1. Yin, T. et al. Pressure-engineered structural and optical properties of two-dimensional $(C_4H_9NH_3)_2PbI_4$ perovskite exfoliated nm-thin flakes. *J. Am. Chem. Soc.* **141**, 1235-1241 (2019).
2. Huang, X. et al. Understanding electron–phonon interactions in 3D lead halide perovskites from the stereochemical expression of $6s^2$ lone pairs. *J. Am. Chem. Soc.* **144**, 12247-12260 (2022).
3. Fabini, D. H. et al. Dynamic stereochemical activity of the Sn^{2+} lone pair in perovskite $CsSnBr_3$. *J. Am. Chem. Soc.* **138**, 11820-11832 (2016).
4. Leng, K. et al. Molecularly thin two-dimensional hybrid perovskites with tunable optoelectronic properties due to reversible surface relaxation. *Nat. Mater.* **17**, 908-914 (2018).
5. Tantardini, C., Kvashnin, A. G., Gatti, C., Yakobson, B. I., Gonze, X. Computational modeling of 2D materials under high pressure and their chemical bonding: silicene as possible field-effect transistor. *ACS Nano* **15**, 6861-6871 (2021).
6. Kühne, T. D. et al. CP2K: An electronic structure and molecular dynamics software package - Quickstep: Efficient and accurate electronic structure calculations. *J. Chem. Phys.* **152**, 194103 (2020).
7. Carignano, M. A., Aravindh, S. A., Roqan, I. S., Even, J., Katan, C. Critical fluctuations and anharmonicity in lead iodide perovskites from molecular dynamics supercell simulations. *J. Phys. Chem. C* **121**, 20729-20738 (2017).
8. Kaiser, W. et al. First-principles molecular dynamics in metal-halide perovskites: contrasting generalized gradient approximation and hybrid functionals. *J. Phys. Chem. Lett.* **12**, 11886-11893 (2021).
9. Delley, B. An all-electron numerical method for solving the local density functional for polyatomic molecules. *J. Chem. Phys.* **92**, 508-517 (1990).
